# Neurosustainability

**DOI:** 10.3389/fnhum.2024.1436179

**Published:** 2024-08-29

**Authors:** Mohamed Hesham Khalil

**Affiliations:** Department of Architecture, Faculty of Architecture and History of Art, School of Arts and Humanities, University of Cambridge, Cambridge, United Kingdom

**Keywords:** neuroplasticity, environmental enrichment, nature, cortical thickness, adult hippocampal neurogenesis, amygdala, mental health and modern lifestyles, human brain health

## Abstract

While the human brain has evolved extraordinary abilities to dominate nature, modern living has paradoxically trapped it in a contemporary “cage” that stifles neuroplasticity. Within this modern environment lurk unseen natural laws with power to sustain the human brain’s adaptive capacities - if consciously orchestrated into the environments we design. For too long our contemporary environments have imposed an unyielding static state, while still neglecting the brain’s constant adaptive nature as it evolves to dominate the natural world with increasing sophistication. The theory introduced in this article aims to go back in nature without having to go back in time, introducing and expounding Neurosustainability as a novel paradigm seeing beyond the contemporary confines to architect environments and brains in parallel. Its integrated neuro-evidenced framework proposes four enrichment scopes—spatial, natural, aesthetic, and social—each holding multifaceted attributes promising to sustain regions like the hippocampus, cortex and amygdala. Neurosustainability aims to liberate the quintessential essence of nature to sustain and enhance neuroplastic processes through a cycle that begins with design and extends through epigenetic changes. This paradigm shift aims to foster cognitive health and wellness by addressing issues like stress, depression, anxiety and cognitive decline common in the contemporary era thereby offering a path toward a more neurosustainable era aiming to nurture the evolution of the human brain now and beyond.

## 1 Introduction

The human brain is ever-changing, and over time, our brains have been evolving to dominate nature with more sophistication. Still, it is uncertain whether, in today’s contemporary world, our brains are evolving in parallel with and through the environments we create. Since early modernity and now in our contemporary era, a significant percentage of people are leaning toward sedentary lifestyles, facing more daily stressors, losing contact with nature, and suffering more from psychological problems such as anxiety and depression. Ignoring the interrelationships between these variables and the human brain is impossible. It is equally impossible to ignore the paradoxical effect of the contemporary built environment. While it contributes to the de-evolution of the human brain, it can be enriched to architect the neuroplastic brain. In that regard, this article introduces Neurosustainability as a paradigm shift beyond the static state of contemporary and modern living that has multiple cul-de-sac effects on the human brain’s ever-changing neuroplasticity. Sustaining the neuroplasticity processes of the human brain is feasible through environmental enrichment, which has been predominantly explored over the past sixty years on non-human subjects with recent interest in translation from rodents to humans. This article introduces and establishes Neurosustainability as a novel and critically needed paradigm shift, holding antidotes for the nature-alienating contemporary environment. Subsequent sections address the current problems in this modern environment before introducing and expounding Neurosustainability.

### 1.1 The human brain in the ‘contemporary cage’

Over time, nature has gradually changed from its pristine and untamed form to a designed and controlled environment by the human brain that evolved through it in the past. However, the evolution of the human brain may not necessarily mean an equal evolution of the environment it is creating when the human brain is in a constant state of neuroplasticity and under the influence of the limited variables it knows. In other words, the deviation from the untamed form of nature to an entirely tamed world by humans has transformed the interrelationship between nature and the human brain’s plasticity from ambiguous top-down to a bottom-up feedback loop with a limited vision. Increasing evidence suggests that modern living environments and lifestyles may negatively impact various aspects of human well-being. We argue that the lost environmental enrichment in urban and built environments holds the antidotes.

For instance, cognitive well-being is deeply influenced by environmental complexity and lifestyle factors. Often characterized by uniformity and predictability, contemporary living environments lack the stimulation necessary for cognitive development and maintenance (Kempermann, 2019). Sedentary lifestyles further contribute to cognitive decline, as physical activity is crucial for maintaining brain health (Macpherson et al., 2017; Di Liegro et al., 2019). Moreover, environmental toxins, such as air pollution and chemicals in household products, have been shown to affect cognitive functions negatively (Clifford et al., 2016; Yuan et al., 2023).

Decreased use of pattern separation in contemporary lifestyles may contribute to hippocampal atrophy (Birkel, 2017), while the rise in mental health disorders, including anxiety, depression, and chronic stress, is a significant concern in the modern world (Rojas-Carvajal et al., 2022; Moitra et al., 2023). Work pressure, financial instability, and personal and relationship conflicts contribute to this increase, putting extra pressure on the environment.

Based on the previous discussion, if rodents are housed in cages with four walls and humans live in an almost open-ended world, that does not mean humans live in more enriched environments than rodents. This is because certain environmental variables contribute to sustaining the different neuroplasticity processes and subsequently improving related functions such as learning, memory, pattern separation ability, navigation, cognitive function, psychological health, and restorativeness. The concept of a “contemporary cage” aptly describes the restrictive and often detrimental impact of modern urban and built environments on the human brain. These meticulously designed settings impose uniformity that limits the brain’s plasticity and adaptability, potentially contributing to chronic stress, cognitive problems, anxiety, depression, sedentary lifestyles, and social isolation. This article introduces the novel concept of Neurosustainability, exploring how spatial, natural, aesthetic, and social enrichment can hold different variables with multi-faceted attributes for the negative impact of the contemporary environment on neuroplasticity.

### 1.2 Neuroplasticity: cerebral cortex, hippocampus, and amygdala

Neuroplasticity is the brain’s ability to reorganize itself by forming new neural connections throughout life, enhancing learning, memory, spatial navigation, emotional regulation, and adaptive responses in the cortex, hippocampus, and amygdala (LeDoux, 2000; Kolb and Gibb, 2015; Squire et al., 2015), and other brain regions.

The cerebral cortex, the brain’s outermost layer, is a highly organized and complex structure responsible for a wide range of cognitive, sensory, and motor functions. It plays a crucial role in perception, language, memory, attention, thought, consciousness, and decision-making. The cortex exhibits remarkable plasticity, which refers to its ability to undergo structural and functional changes in response to experiences, learning, and environmental factors throughout an individual’s lifespan (Kolb and Gibb, 2015). Structural plasticity measures, such as cortical thickness, gray matter volume, and white matter integrity, can be assessed using neuroimaging techniques like magnetic resonance imaging (MRI) and diffusion tensor imaging (DTI) (Tardif et al., 2016). Functional plasticity, on the other hand, can be evaluated using functional MRI (fMRI), electroencephalography (EEG), and transcranial magnetic stimulation (TMS), which provide insights into the brain’s ability to reorganize its neural networks and adapt its activity patterns in response to various stimuli and experiences (Pascual-Leone et al., 2005).

The hippocampus plays a vital role in learning, memory formation, and spatial navigation (Squire et al., 2015). The hippocampus exhibits remarkable structural and functional plasticity, enabling it to adapt and reorganize in response to various experiences and environmental factors (Leuner and Gould, 2010). Structural plasticity processes in the hippocampus include neurogenesis, the formation of new neurons in the dentate gyrus throughout adulthood (Kempermann et al., 2015), dendritic remodeling, changes in the morphology and complexity of dendritic arbors influenced by experience and learning, and synaptic plasticity, the strengthening or weakening of synaptic connections between neurons, which is crucial for information processing and storage. The hippocampus also contains place cells, pyramidal neurons that fire selectively when the subject is in a specific location within an environment (O'Keefe and Dostrovsky, 1971). Place cell activity is influenced by various factors, including sensory cues, goal locations, and past experiences (Poucet and Hok, 2017).

The amygdala, located deep within the medial temporal lobe, plays a crucial role in emotional processing, fear conditioning, and memory formation (LeDoux, 2000). It has extensive connections with various brain regions, including the prefrontal cortex, hippocampus, and sensory areas (Janak and Tye, 2015). The amygdala is involved in the acquisition, storage, and expression of fear memories, as well as in the modulation of attention, perception, and decision-making processes related to emotional stimuli (Phelps and LeDoux, 2005). Structurally, the amygdala undergoes plasticity in response to emotional experiences, with changes observed in dendritic arborization, spine density, and neuronal morphology (Vyas et al., 2002; Mitra et al., 2005). Stress has been shown to significantly impact the structure and function of the amygdala, with chronic stress leading to dendritic hypertrophy and increased spine density in the basolateral amygdala, which may contribute to heightened anxiety and fear responses (Vyas et al., 2002; Roozendaal et al., 2009). Understanding these effects is crucial for regulating neuroplasticity through the environment.

After exploring the neuroplasticity nature of the cerebral cortex, hippocampus, and amygdala, it is crucial to understand how environmental factors influence these brain regions. The following sections discuss epigenetics and environmental enrichment, initially conducted on rodents, and its potential implications for human brain plasticity in contemporary living environments where there might be a lack of human-based neuro-evidences at specific points.

### 1.3 Epigenetics and BDNF: the process of change

In addition to the challenges posed by modern living environments on cognitive well-being and mental health, it is crucial to consider the role of epigenetics in mediating environmental factors’ effects on brain health and neuroplasticity. A plethora of external stimuli collectively referred to as ‘epigenetic factors’ strongly influence the brain’s structural and functional reorganization, thereby acting as a potential driver of neural plasticity (Nayak et al., 2022). Epigenetics is the study of heritable changes in gene expression that occur without alterations in the underlying DNA sequence (Champagne, 2010). These changes are mediated by various epigenetic mechanisms, such as DNA methylation and histone modifications, which can influence the accessibility and transcription of genes.

Epigenetic mechanisms, with their dynamic nature, play a critical role in brain development, plasticity, and function. Sweatt (2013) demonstrated that epigenetic modifications are essential for long-term memory formation, suggesting a critical link between epigenetic regulation and neuroplasticity. The dynamic regulation of DNA methylation patterns in the brain during development and in response to environmental factors, such as stress, diet, and social interactions (Champagne, 2010), underscores the ever-changing nature of the brain. Histone modifications have been implicated in the regulation of synaptic plasticity, learning, and memory (Fischer et al., 2007).

The environmental factors addressed in the Neurosustainability framework may affect the brain partly through epigenetic modifications. Exposure to enriched environments has been shown to induce epigenetic changes in the brain, leading to altered gene expression profiles and enhanced neuroplasticity (Mychasiuk et al., 2012). For example, environmental enrichment has increased histone acetylation levels in the hippocampus, promoting gene expression in synaptic plasticity and cognitive function (Fischer et al., 2007). The multifaceted variables of nature discussed in the Neurosustainability framework may induce epigenetic changes in these brain regions, promoting neuroplasticity and brain health.

Recent research suggests that the expression of Brain-Derived Neurotrophic Factor (BDNF), a protein that plays a vital role in neuronal growth, survival, and synaptic plasticity, can be modulated by epigenetic mechanisms, such as DNA methylation and histone modifications, which are influenced by environmental factors. Environmental enrichment, which is to be discussed shortly, has been shown to modulate BDNF expression in humans through epigenetic mechanisms, such as DNA methylation and histone modifications. Recently, it was found that environmental enrichment, such as social contact, exposure to novelty, tactile stimuli, and voluntary exercise, increases BDNF transcripts in the prefrontal cortex, suggesting an environmental enrichment-induced epigenetic control of BDNF expression (Costa et al., 2023). This appears to be highly relevant to the discourse in this article as patients diagnosed with psychiatric disorders show decreased neural BDNF levels, often associated with increased DNA methylation at specific BDNF promoters (Ikegame et al., 2013). Additionally, hippocampal epigenetic modification at the BDNF gene is induced by environmental enrichment (Kuzumaki et al., 2011). Furthermore, DNA methylation and DNA methyltransferases regulate neurogenesis through epigenetic control of gene expression, while neurogenesis is associated with psychiatric disorders and BDNF levels (Yao et al., 2016).

These findings suggest that epigenetics and the epigenetic modulation of BDNF expression may play a crucial role in mediating the beneficial effects of environmental enrichment on brain plasticity and cognitive function, with potential implications for the development of novel therapeutic strategies for neurological and psychiatric disorders. Given the growing evidence for the role of epigenetics in brain health and neuroplasticity, it is essential to delve deeper into the understanding of how the variables and attributes of the Neurosustainability framework may induce epigenetic changes that contribute to the maintenance of brain function in the face of complex environmental factors. By gaining a comprehensive understanding of the epigenetic mechanisms underlying the benefits of enriched environments on the brain, we can develop targeted interventions to promote lifelong brain health and resilience.

### 1.4 Environmental enrichment from rodents to humans

Research on environmental enrichment on humans is limited due to methodological limitations and assumptions that human environments are inherently complex and stimulating. As contemporary humans, we may have never come to criticize our creature, the contemporary built environment, for how ineffective it is for sustaining our brains. However, recent studies comparing the living environments of rodents and humans suggest that the complexity of human environments may be overestimated, warranting a closer examination of the translational potential of rodent research findings. This section reviews the existing body of knowledge on environmental enrichment interventions that positively affect rodent brains and discusses translation from rodents to humans to use to build Neurosustainability whenever a shortage is found regarding human-based studies for any variable.

One of the earliest studies revealing this interrelationship between environmental complexity and neuroplasticity was by Krech et al. (1960), who found in their study that the ratio of cortical to subcortical cholinesterase activity was subject to environmental complexity, which means that the brain adapts differently to complex environments, with the cortex potentially undergoing more significant alterations in response to increased cognitive demands. Another crucial early study was published by Diamond et al. (1964), who found that the cerebral cortex of rats subjected to training showed not only an increase in acetylcholinesterase, and enzyme that breaks down the neurotransmitter acetylcholine, which is essential for learning, memory, and muscle function, but also an increase in volume. These studies show that environmental enrichment not only changes functional plasticity but also changes the brain structure.

Another vital brain structure is the hippocampus, which is associated with learning and memory, spatial navigation, pattern separation ability, place cell formation, and remapping, among other essential functions (Bird and Burgess, 2008; Sahay et al., 2011; Fenton, 2024). Some of the earliest studies where different strains of mice were housed in laboratory cages have revealed that more neurons were found in the dentate gyrus of the hippocampus after exposure to the environment (Kempermann et al., 1997, 1998a). This process is a structural form of plasticity in the hippocampus represented by the birth of new neurons. Neurogenesis usually decreases with aging, but Kempermann et al. (1998b) have found that exposure to environmental enrichment had positive results. Another study later confirmed that environmental cues can enhance neurogenesis in the adult hippocampal region (Nilsson et al., 1999). As more studies came out, it became evident that environmental enrichment is dependent on multifaceted complexity responsible for increasing neurogenesis and synaptic plasticity (Segovia et al., 2006; Hattori et al., 2007; Leal-Galicia et al., 2008; Leger et al., 2015; Hüttenrauch et al., 2016). This area of research has matured, with over forty years of research present (Fuchs and Flügge, 2014).

One study on rodents revealed that artificial enrichment may affect the brain differently than natural stimuli, which were associated with enhanced environmental interactions (Lambert et al., 2016). The study revealed less activation of the c-fos gene in the amygdala following a water escape task, suggesting that environmental enrichment (natural and artificial) may have reduced the stress response or fear-related activity in this brain region. However, increased c-fos activation was observed in the nucleus accumbens, specifically in the natural-enriched animals, indicating that exposure to a more natural environment may have enhanced reward processing or motivation. Hence, environmental complexity *per se* is multifaceted, and its interrelationships with neuroplasticity are multidimensional yet missing in our contemporary world, where we have evolved to transform the untamed nature into sophisticated cities and crafted micro cages within cities.

Here, the rodent-reliant discourse can help escape the cul-de-sac of contemporaneity. On the one hand, Clemenson et al. (2015) have reviewed the potential impact of environmental enrichment without running wheels on neurogenesis, drawing comparisons between evidence from mice and findings from studies on humans, suggesting that neurogenesis, pattern separation ability and other hippocampal plasticity outcomes are more similar than different between mice and humans. On the other hand, a couple of articles compared both living environments of rodents and humans, highlighting limitations and potential translation (Kempermann, 2019; Rojas-Carvajal et al., 2022). Most recently, a systematic review aimed to understand the nuanced differences of spatial environmental enrichment interventions on rodents, finding that the degree, diversity, and change of complexity along with space size, navigational novelty, and physical activity levels are all factors contributing to improving hippocampus plasticity and neurogenesis (Khalil, 2024). That review provides a translational model for humans by highlighting that it might be the contemporary environment of humans that is impoverished compared to rodents.

By untangling nature and expounding the multifaceted nature of its variables, environmental enrichment of built environments can benefit from the quintessential essence of nature to sustain the neuroplasticity processes of the human brain. The following sections deconstruct environmental enrichment and expound Neurosustainability through four types of enrichment, as well as variables and multifaceted attributes that affect the cortex, hippocampus, and amygdala neuroplasticity processes uniquely.

## 2 Neurosustainability

**Figure 1:**
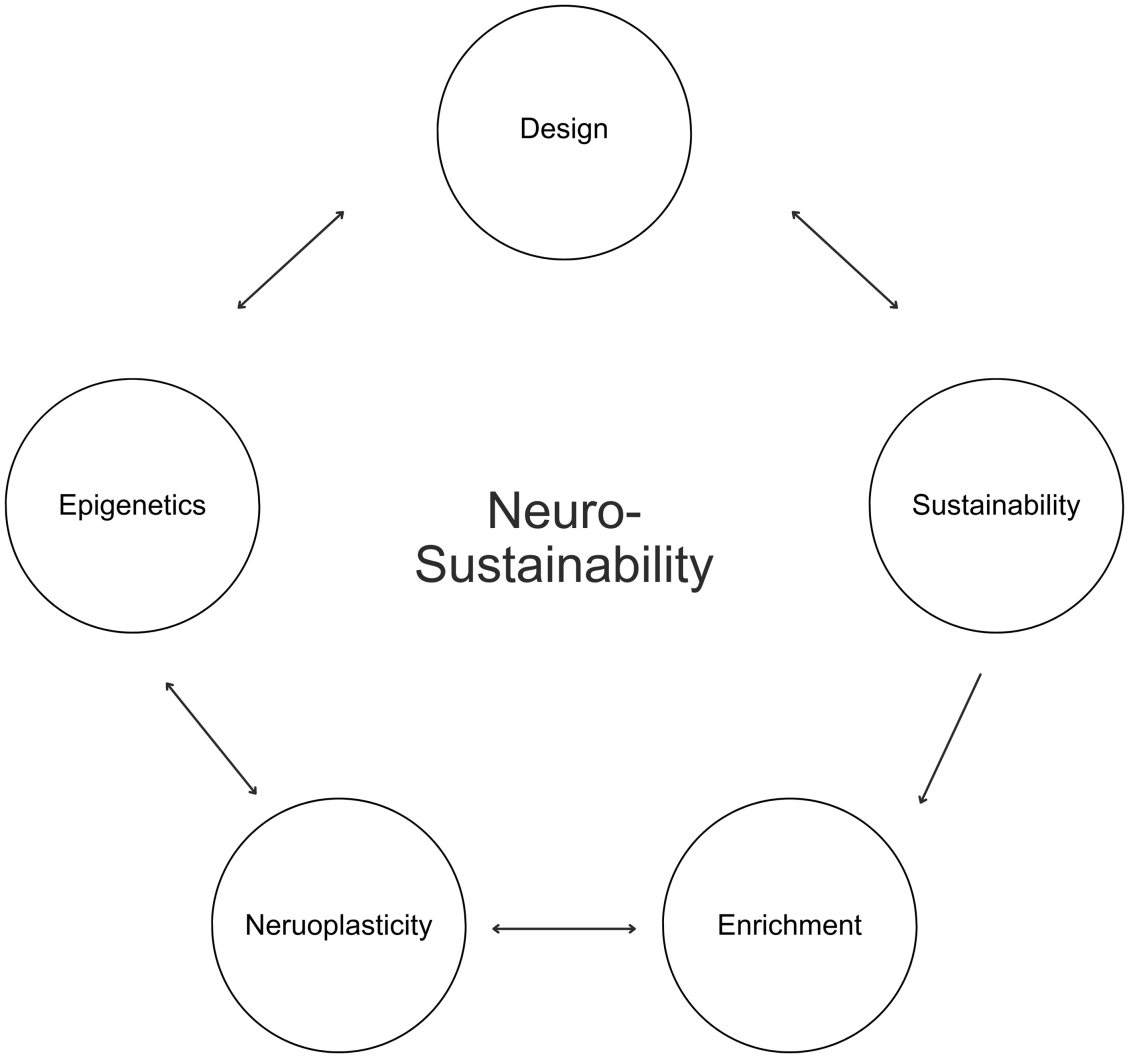
Neurosustainability.

This article introduces Neurosustainability, as shown in Figure 1, as a novel theory, which encompasses an ongoing cycle beginning with the design of built, urban, architectural, and interior environments and their role towards a sustainable neuroplasticity through long-term epigenetic changes. This theory posits that sustainable practices should consciously consider the different variables in nature that possess distinct attributes, and how these enrichment variables, whether spatial, natural, aesthetic, or social, affect neuroplasticity in various brain regions in humans, including the hippocampus, cortex and amygdala. Furthermore, this article argues that this process takes place through epigenetics, and that long-term epigenetic changes in humans not only can facilitate the neuroplastic changes but also influence how humans can inherit improved brain blueprints and perceive and design their environments in the future for an ongoing sustainable neuroplasticity, ultimately capitalizing on the essence of our natural environments beyond contemporary living.

**Figure 2:**
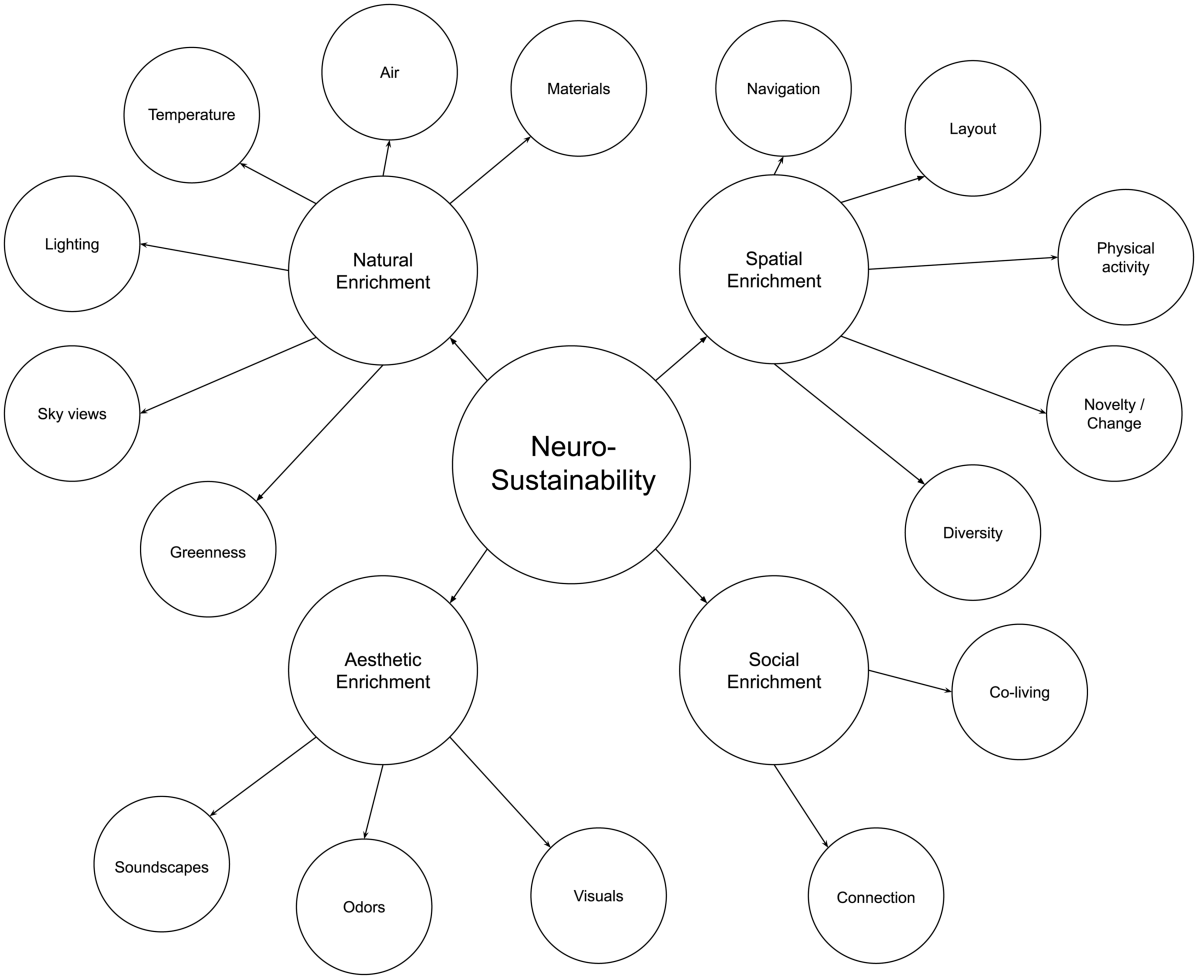
The neurosustainability framework.

Afterward, this article expounds on Neurosustainability through the breakdown of enrichment into spatial, natural, aesthetic, and social. Afterward, this article discusses the variables for each group of enrichment, as shown in Figure 2, before the paper continuous with showing the multifaceted attributes of each variable on the cortex, hippocampus, and amygdala and how the knowledge about those factors can provide a paradigm shift with more intersection of variables and less divergence in disciplines to facilitate long-term Neurosustainability.

### 2.1 Neurosustainability through spatial enrichment

**Figure 3:**
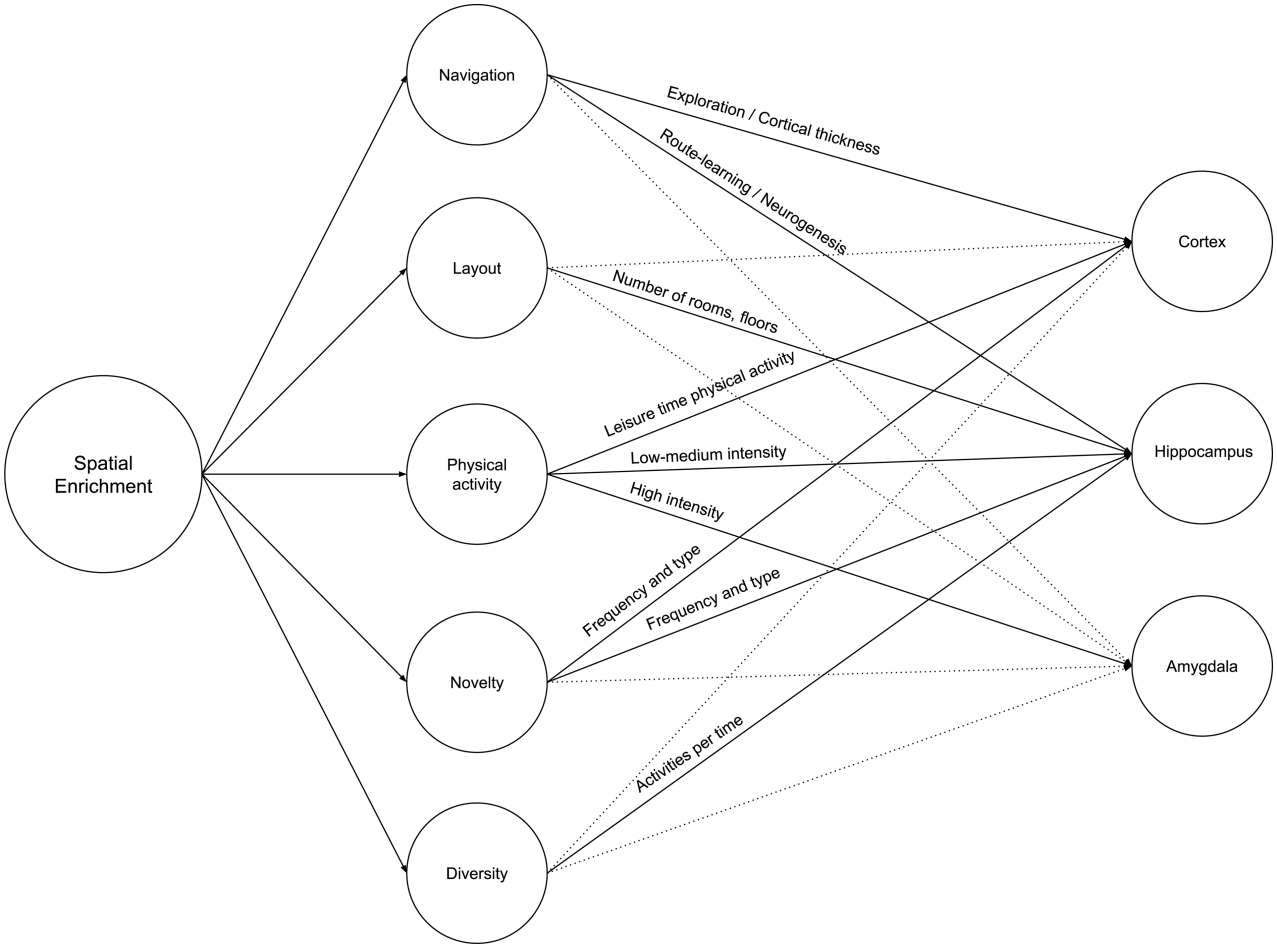
Neurosustainability through spatial enrichment: variables and attributes.

Spatial enrichment is a growing concern at both the urban and architectural levels. At the urban level, it is defined as the quantified geospatial properties (road network and landmark elements) (Shin et al., 2024), while at the architectural level, it is defined as the building’s layout complexity (Coburn et al., 2020). Both concepts were compared to cage models by Khalil (2024), such as the Marlau cage and the Hamlet Complex Maze, which emphasizes that spatial enrichment is similar than different between rodents and humans who share similar neurogenesis characteristics as reported by Clemenson et al. (2015). This article argues that the impact of increased spatial enrichment is multifold. However, the definition of spatial enrichment *per se* is multidimensional: Increased navigation and space exploration, increased layout complexity, increased through-space physical activity, increased change and novelty, and increased diversity of activities. This section explores the attributes of those multifaceted variables and each variable’s effect on the cortex, hippocampus, and amygdala. This should help future research to control and quantify variables accordingly and to maximize the prospective impact of spatial enrichment beyond a sole brain region or structure. Figure 3 illustrates the summary of Neurosustainability through spatial enrichment before expounding it in detail.

#### 2.1.1 Navigation

Navigation is one of the most explored spatial enrichment-related variables on human subjects. Regarding the cerebral cortex, Wenger et al. (2012) have investigated the age difference in cortical thickness changes, a marker of structural plasticity, evoked by spatial navigation training in a complex environment where spatial navigation training resulted in cortical thickening in the left precuneus and paracentral lobule in young navigators only. The precuneus is a brain region located in the medial parietal lobe, which is involved in various cognitive processes, including visuospatial processing, episodic memory, and self-referential processing (Cavanna and Trimble, 2006), while the paracentral lobule is a brain region located at the medial surface of the cerebral hemispheres, which is involved in motor and sensory functions, particularly those related to the lower limbs (Frigeri et al., 2015). This is the only study found on human subjects regarding cortical plasticity in response to spatial complexity and navigation.

However, while there is a need for more research on the impact of spatial complexity-dependent navigation on cortical plasticity, there is a plethora of research already on the hippocampus. The earliest studies were done on London taxi drivers who were found to have structural changes in their hippocampus due to navigation-related variability. The earliest study has revealed that the hippocampus is involved in processing spatial layouts established over long-time courses, highlighting that the right hippocampus is recruited in large-scale spatial environments (Maguire et al., 1997). Afterward, another study revealed that the hippocampi of taxi drivers were significantly larger relative to subjects who did not drive taxis, highlighting structural hippocampal plasticity in response to environmental demands (Maguire et al., 2000). A few years later, it was revealed that London taxi drivers’ extensive navigation experience is associated with greater gray matter volume in the mid-posterior hippocampus and less volume in the anterior hippocampus compared to bus drivers, suggesting that spatial knowledge, rather than stress or driving, is linked to hippocampal structural differences and may come at a cost to acquiring new spatial memories (Maguire et al., 2006). Since then, there has been an increasing interest in the topic till now (Griesbauer et al., 2022).

While those studies prove the translation of spatial enrichment paradigms from rodents to humans, whether pedestrians can benefit from the exact attributes remains to be seen. Two studies have explored the impact of cognitively demanding spatial navigation tasks on the hippocampus for adults as well as elders, finding that changes in demands on spatial navigation can alter hippocampal N-acetyl aspartate (NAA) concentrations, a crucial neuronal marker that plays a role in neural metabolism, with the BDNF genotype acting as a moderator for these plastic changes (Lövdén et al., 2011), and that the hippocampal barrier density increased in density followed by a return to baseline in the right hippocampus, which we previously evidenced to be active with London taxi drivers navigating large scale complex environments (Maguire et al., 1997), but declined in the control group and the left hippocampus, suggesting that cognitively demanding spatial navigation is beneficial for both adults and elders (Lövdén et al., 2012). Those studies took place for four months, which challenges the effectiveness of spatial navigation on the hippocampus in short-term interventions. However, a study by Keller and Just (2016) examined whether neuroplasticity results from learning spatial routes for 45 min, revealing decreased diffusivity in the posterior-dorsal dentate gyrus of the left hippocampus in the route learning group, accompanied by fMRI-measured blood-oxygen-level-dependent (BOLD) signals between this region and cortical areas. Decreased diffusivity in the dentate gyrus of the hippocampus suggests that there may be increased cellular density, myelination, or synaptic complexity in this region. This change is often interpreted as a sign of structural plasticity and adaptation in response to learning. Since it is known that pattern separation ability is a function of the hippocampus positively correlated with neurogenesis, Kolarik et al. (2020) have revealed that spatial exploration improves hippocampal-dependent memory in older adults who scored better on the lure discrimination index (LDI). Last but not least, Shin et al. (2024) found that greater geospatial environmental complexity was associated with larger allocentric (but not egocentric) navigation-related brain volumes across the Alzheimer’s disease spectrum. Allocentric regions include the hippocampus, the parahippocampal gyrus, and the entorhinal cortex. The latter projects to and receives significant connections from the hippocampus. Navigation is also an important variable that affects the hippocampal synaptic plasticity. Priestley et al. (2022) found that behavioral-timescale synaptic plasticity (BTSP) was better during novel context exploration and decayed with experience, stabilizing within minutes of the first exposure to a new environment. It is essential to highlight that hippocampal plasticity requires an additional post-exposure phase, which is sleep. For instance, Deantoni et al. (2021) have investigated sleep-related changes after navigation learning and relearning, finding that memory traces were less efficiently consolidated after post-training sleep deprivation, unlike participants who had a regular sleep, which is also confirmed by Villemonteix et al. (2023) who found that navigational training sleep-related changes were found at day 4 in hippocampus structures.

The limitation on that variable, however, remains in the lack of exploring the impact of navigational complexity in built environments on both the hippocampus and the cortex, especially in living spaces such as houses where people tend to be more sedentary, and spaces are juxtaposed with minimal transitional spaces, corridors, and sometimes are in an open plan. Another limitation here lies in the unexplored impact of navigation on the amygdala at both the urban and built environment scales to test whether it induces stress or not. It is hard to rely on rodent studies here because stress and behavior can vary between rodents and humans.

#### 2.1.2 Layout

We distinguish between navigation and layout complexity in the built environment as the former is represented by the complexity of corridors, staircases, and pathways, while the latter is represented by the presence of multiple rooms and multiple floors. Though very little is known about whether increasing the layout complexity of the built environment can improve neuroplasticity the same way as navigation on the urban scale does, we provide initial support for this hypothesis through rodent-based studies that were found to have better effects on hippocampal neurogenesis by increasing the layout complexity, which was argued by Khalil (2024) to be translatable for human housing conditions such as multi-story and courtyard houses.

#### 2.1.3 Physical activity

It is already established that physical exercise is proven to have positive effects on the human hippocampus (Firth et al., 2018). We argue that the sole reliance on physical exercise as an antidote for the sedentary behaviors induced by the built environment overshadows the shortcomings of the modern and contemporary built environment. One study has reported that weekend/holiday sedentary behavior and the lack of physical activity, as we hypothesize here, were associated with fractional anisotropy but not with gray matter volume (Pineda et al., 2021). In this section, physical activity is introduced as an independent variable yet can be influenced by the increase of navigation or layout complexity.

Research done on humans proves that the more significant amount, duration, and frequency of total daily walking activity are all associated with larger hippocampal volume, but among women, not men (Varma et al., 2015), while physical activity in general, in the form of gardening activity, was found by Park et al. (2019) to have positive effects on the BDNF and PDGF levels among elders. Through rodent studies, this article supports that the combined effect of physical activity and spatial complexity on neurogenesis to expound that both variables have an additive effect. As the Marlau cage’s complexity was proven to affect AHN due to navigation and navigational novelty (Fares et al., 2013), Santoso et al. (2020) have revealed that combined physical activity with the Marlau cage had the highest levels of hippocampal BDNF and NGF levels, indicators of neurogenesis as well. Hence, we argue that walking and daily step count can be influenced by increased layout complexity and navigation, and consequently, it needs to be quantified accordingly. This can apply not only to the built environment but also to urban environments. Existing research that objectively quantified physical activity and sedentary behaviors in children and associated the variability with hippocampal gray matter volume (Migueles et al., 2020) and used a wearable device to associate physical activity with gray matter volume (Domingos et al., 2021a), can be considered for guidance.

Regarding the amygdala, little is known about variables that affect its plasticity. However, it is argued whether walking itself or walking in the forest is what helps reduce cortisol levels, as evidenced by psychological indicators and salivary cortisol biomarkers (Kobayashi et al., 2017, 2019, 2021). From a neurological perspective, it was found that running elicited stronger amygdala reactivity to implicit happiness than fear, but it was found that walking did the opposite (Chen et al., 2019). Additionally, higher levels of cardiorespiratory fitness were associated with the expansion of the right amygdala (Cadenas-Sanchez et al., 2023). The results suggest that habitual physical activity could mediate acute exercise-induced anxiolytic effects concerning amygdala reactivity. The scarcity of results still does not disqualify walking as a positive influencer on the amygdala, and more research is needed.

Regarding the cortex, a recent systematic review has highlighted that higher activity levels have been linked to larger volumes of brain gray matter in areas such as the hippocampus and prefrontal cortex, as well as to maintained white matter integrity, fewer severe white matter lesions, and enhancements in white matter microstructure (Domingos et al., 2021b). Another study supports that individuals who reported more leisure time physical activity had larger brain volume and cortical thickness than those who reported less leisure time physical activity (Gu et al., 2020). Falck et al. (2020) and Sun et al. (2024) have found in their studies that there is a positive relationship between physical activity, as a lifestyle-related variable, and cortical thickness.

Built environments should be designed to encourage physical activity with or without spatial complexity to promote brain health. Future research should investigate the impact of navigational complexity on the amygdala and cortex and explore the potential benefits of combining physical activity with spatially complex environments.

#### 2.1.4 Novelty

Change and novelty, while they can be considered as facets of navigation, can also add more depth to the definition when they are related to interior complexity inside the space. This area of research is still immature, but a very recent review by Khalil (2024) identified how changing navigation or changing in-space complexity has an additive effect on adult hippocampal neurogenesis (AHN) independently from the effect of physical activity and navigation. The difference between interior complexity for rodents and humans makes it hard to expect how novelty in interior spaces can be quantified. Nevertheless, the distinctiveness of the variable *per se* must be considered. As reported by Khalil (2024), the majority of studies focus on changing in-space complexity by interchanging or replacing elements with unique characteristics, even in short-term studies, and it was proven to be successful in inducing neurogenesis even with the exclusion of running wheels yet resulted in longer durations of exposure. However, no hippocampal neurogenesis was observed with a single structural element of enrichment (a red acrylic tunnel) without running wheels or complex environmental enrichment. The diversity of interior complexity, the presence of cognitively demanding objects, and the frequency of change may be further explored. There is evidence that homebodies who spend more time in an unchanging environment, unlike others who venture into the city, have poorer cognitive functions (McCormick et al., 2022). Spatial experiences requiring exploration have encompassed novelty that was also proven to be effective for the hippocampus and cortical plasticity as evidenced by Wenger et al. (2012).

#### 2.1.5 Diversity

Diversity, which we can expound as a high frequency of different activities experienced within a fixed duration, appears as another critical variable for increasing hippocampal volume and that can be considered at both the urban and built environment levels through the presence of multiple commercial/public space uses and diverse space uses, respectively.

The problem of lacking diversity was reported by Folville et al. (2023), who revealed that during lockdown, there were changes in experiential diversity that negatively affected well-being. On the contrary, Urban-Wojcik et al. (2022) have tested whether greater activity diversity, defined as the range of common daily activities engaged in and the proportion of time spent in each, is associated with larger hippocampal volume, finding a positive correlation across the left and right hemispheres. This study provides neurological evidence to an earlier study by Lee et al. (2021), who found that greater activity diversity was associated with overall cognitive functioning, higher executive functioning, and episodic memory. Another study confirms that greater diversity in daily activities was related to higher cognitive functioning after adjusting for physical activity (Brown et al., 2023).

Interestingly, one possible explanation is that engaging in various activities may provide attention and challenging experiences, which can stimulate the formation of new neural connections and promote the survival of newly generated neurons in the hippocampus. Additionally, diverse experiences enhance the complexity and richness of the neural representations in the hippocampus, which could support better memory formation and retrieval.

### 2.2 Neurosustainability through natural enrichment

**Figure 4:**
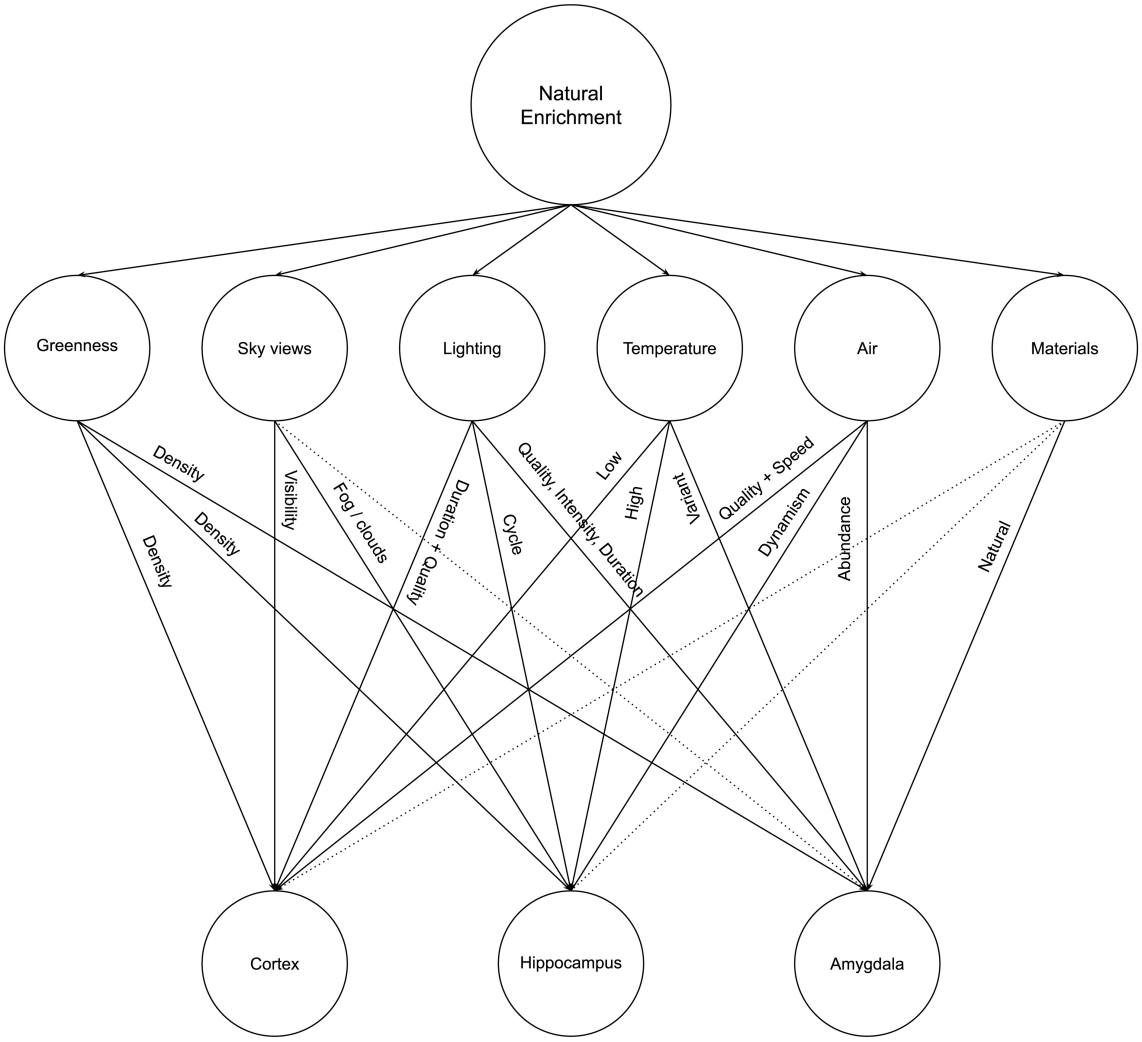
Neurosustainability through natural enrichment: variables and attributes.

The lack of greenness is one of the most explicit and common criticisms of urban and built environments, which has led to the increasing interest in residential greenness for neighborhoods and urban environments to promote well-being (Son et al., 2021) and in biophilia for architecture and interiors as a promoter for health, well-being, and sustainability (Huntsman and Bulaj, 2022; Zhong et al., 2022). In this article, we argue that Neurosustainability is a complex process that can affect the human brain at multiple levels and through different multifaceted variables: Tree cover density and greenness, sky views, lighting, temperature, air, and materials. Figure 4 illustrates a summary of Neurosustainability through natural enrichment before each variable is expounded subsequently.

#### 2.2.1 Greenness

A plethora of studies support a strong and significant relationship between cortical plasticity and residential greenness that is defined by tree cover density. Firstly, Kühn et al. (2023a) have investigated the association between tree cover density (500 m buffer around the home) and brain structure, observing a positive association between tree cover density and gray matter volume in the right orbitofrontal cortex. Secondly, Min et al. (2021) have assessed the association between residential greenness as well (750 m buffer) and cortical thickness among late adulthood and elderly, also finding that it is positively correlated with the cortical thickness of global, parietal, and occipital regions. Interestingly, the association was in urban populations, not rural populations. Thirdly, Kühn et al. (2021) have also investigated the structural and functional neural basis of urbanicity and green space (1 km buffer around the home), finding a negative association between the urban fabric and gray matter volume in contrast with a positive association in urban green and gray matter volume in the pregenual/subgenual anterior cingulate cortex, a part of the prefrontal cortex located in the medial wall of the cerebral hemispheres. Fourthly, this relationship among elders was further supported by a third study by Besser et al. (2021), who investigated the associations between neighborhood greenspace and MRI measures, findings that there is a possible association between the abundance of green space and less ventricular enlargement, suggesting a minor deviation from the normal ventricular size compared to what is typically expected. In other words, more green space is associated with less global brain atrophy. Deprivation from green spaces is found to be a risk factor for worsening white matter grade as well (Besser et al., 2023).

Little is known about the impact of greenness or tree cover density on the hippocampus except through a recent study that revealed that a walk in the forest was found to have a significant impact on increasing the subiculum volume, a hippocampal subfield involved in stress response inhibition, while no change was observed after the urban walk (Sudimac and Kühn, 2024). We can relate this to the amygdala since the dependent variable is related to stress responses. Regarding amygdala plasticity, Kühn et al. (2017) have tested the effects of forest, urban green, and various variables within the buffer radius around the home on brain structures, finding a significant positive association between the coverage of forest, not urban green, and amygdala integrity. Two additional studies have explored the changes in stress-related brain regions as an effect of walking in natural versus urban environments (Sudimac et al., 2022; Sudimac and Kühn, 2022), finding that the amygdala activity decreased after the walk in nature, but only in women, suggesting that women may benefit more from salutogenic effects of nature. The findings of these studies provide valuable insights into the impact of environmental experiences on amygdala plasticity. They suggest that exposure to natural environments, particularly forests, may positively influence amygdala structure and function, potentially promoting resilience to stress and emotional well-being. The observed gender differences in the short-term effects of nature exposure on amygdala activity highlight the need for further research to understand the underlying mechanisms and potential implications for mental health interventions. It is important to note that these studies provide sufficient evidence to challenge how urban and built environments are deviated from the innate essence of nature.

#### 2.2.2 Sky views

Interestingly, and while still part of the overarching theme of residential greenness, one study distinguishes between residential greenness as green space (trees) versus open green space (associated with visibility of the sky) where Kühn et al. (2023b) have observed a positive association between gray matter volume in various prefrontal clusters and green spaces (200 m buffer around home) associated with visibility of the sky, but a negative relationship with prefrontal clusters for tree cover density alone among children. The sky’s visibility was the most critical predictor of gray matter volume in the medial prefrontal cortex. The added value of this study contrasts an earlier study on children that did not separate trees from the visibility of the sky that found that exposure to greenness was positively associated with gray matter volume in both the left and right prefrontal cortex and in the left premotor cortex and with white matter volume in the right prefrontal region, in the left premotor region, and both cerebellar hemispheres (Dadvand et al., 2018).

Another factor in the sky that might be related to the hippocampal place cells firing is the presence of fog. Shin et al. (2022) investigated how environmental changes affect memory formation and modification in the hippocampus. By recording place cells in CA1 and CA3 regions of the hippocampus in a virtual reality environment, they found that CA3 place cells are more resistant to individual landmark changes but undergo significant changes to encode distinctly different environments. In contrast, when visual noise (virtual fog) is introduced to a visually rich environment, CA1 place cells split into two groups: one maintaining their field locations while adjusting firing rates to reflect sensory changes, and the other exhibiting global remapping in response to contextual changes. CA3 place cells, however, mainly exhibit rate remapping under the same conditions. These findings suggest that CA1 can simultaneously represent multiple maps of the same environment when subtle visual noise induces sensory and contextual changes.

#### 2.2.3 Lighting

Lighting is a complex and multidimensional natural variable that the built environment needs to apprehend carefully not only as a variable that can save energy (Olajiga et al., 2024) but because daylight *per se* is a source of well-being (Baker and Steemers, 2019), and it does so in a complex way. Light has more variables to consider besides the source, such as the duration of exposure and the cycle of change related to circadian rhythm. Those three variables have different effects on different parts of the human brain through the unique neuroplasticity processes corresponding to the function of each brain structure.

Firstly, Bumgarner et al. (2023) have demonstrated that exposure to artificial light at night, not as a source of artificial light but as a disruptor of the circadian rhythm, has resulted in an altered hippocampal vascular structure and connectivity in mice. This is not the only study reporting that disruption to the natural lighting cycle has adverse effects on the hippocampus plasticity, neurogenesis, and its associated functions such as depression and cognitive performance (Fujioka et al., 2011; Walker et al., 2020; Schröder et al., 2023). However, there is no research on that matter on human subjects who experience more exposure to artificial light of diverse types.

Secondly, regarding the cortex, Kühn et al. (2022) have reported that time spent outdoors among adults is positively associated with gray matter volume in the right dorsolateral prefrontal cortex, noting that the number of hours was a significant predictor, as well as sunshine duration. However, it was found in another study that prolonged exposure to natural sunlight is negatively associated with the volumes of total brain, white matter, gray matter, and white matter hyperintensities (Li et al., 2024). The association between sunlight exposure and brain structure was different for individuals with less than 2 h of daily sunlight exposure compared to those with more than 2 h of exposure. However, it might be a limitation that the group that had exposure to more than 2 h consisted mainly of older people, more likely consisting of males, engaged in higher levels of physical activities and appropriate sleep duration. Furthermore, the authors have highlighted multiple limitations of the recruited participants, which should be considered.

While exposure duration matters most for cortical plasticity, unlike the lighting cycle that matters most for hippocampal plasticity, light quality is also another factor that was reported to be associated with cortical plasticity. For instance, poor lighting was found to be associated with a thinner prefrontal cortex (Uy et al., 2019).

Thirdly, regarding the amygdala, Li et al. (2024) have also reported that sunlight exposure duration was negatively associated with amygdala volume. For the amygdala, another quality of light exposure matters, which is the intensity of light where Campbell et al. (2024) found that as light intensity increased, the activity in several subregions of the amygdala decreased linearly during emotional processing. This suggests that light can modulate the responsiveness of the amygdala to emotional stimuli. Light quality also matters for the amygdala, as evident in exceptional cases. For instance, morning light may improve traumatic stress by reducing reactivity in the amygdala (Cenkner et al., 2022), suggesting that exposure to brighter light conditions can lead to decreased amygdala reactivity compared to dim light settings as well as that exposure to warm light with a higher proportion of long-wavelength energy, specifically at a color temperature of 2,800 Kelvin, seemed to have a suppressive effect on the activity of the amygdala (McGlashan et al., 2021).

However, limited research has been conducted to examine the effects of light therapy on amygdala activity over time. In one notable study, 30 healthy male subjects underwent a 3-week morning white light treatment, with daily 30-min sessions at intensities ranging from 100 to 11,000 lux. The results showed that higher light intensities were associated with more significant reductions in amygdala reactivity from pre- to post-treatment, as measured by an emotional-faces fMRI task (Fisher et al., 2014). These findings provide preliminary evidence to support the notion that morning light therapy may have the potential to alleviate traumatic stress by reducing amygdala reactivity. To further support this hypothesis, Killgore et al. (2022) have found that daily morning blue light treatment (BLT) in post-traumatic stress disorder (PTSD) patients was linked to better objective sleep duration and increased left amygdala volume compared to amber placebo light. Changes in amygdala volume were associated with subjective sleep improvements. These results indicate that morning BLT could be a valuable non-pharmacological addition to PTSD treatment, aiding in sleep and neurobehavioral recovery. In general, individuals with PTSD tend to have smaller amygdala volumes compared to healthy controls. This reduction in amygdala volume has been associated with the chronic stress and hyperarousal experienced in PTSD. Therefore, increasing amygdala volume following blue light therapy could be interpreted as a normalization or recovery of the amygdala rather than a detrimental change.

The research by Majrashi et al. (2022) is a significant contribution to our understanding of the relationship between seasonal changes in photoperiod and amygdala volume. Their study, conducted with 10,033 participants from the UK Biobank cohort, demonstrates the significant effects of photoperiod on amygdala subregion volumes across both hemispheres. Although the study found no direct link between photoperiod-induced amygdala volume changes and mood variations, it provides novel evidence supporting the role of photoperiod in brain structural plasticity, particularly in the amygdala, which is crucial for emotion and cognition. This research proves that light duration is also associated with amygdala plasticity.

#### 2.2.4 Temperature

Temperature is found to be significantly related to both the cortex and hippocampus. Based on the study by Pavlov et al. (2016), lower air temperatures are better for cortical activity, as an increase in air temperature results in a decrease in the activity rate of the right occipital lobe, which suggests that higher air temperatures are not better for cortical activity. Therefore, lower air temperatures would be better for maintaining higher cortical activity. We support this with another study by Lang et al. (2022), who conducted a climate chamber simulation experiment (neutral temperature was set at 26°C and the high indoor temperature at 39°C), finding that the high temperature may damage cognitive performance from the physiology viewpoint.

Seasonal variation, precisely the number of sunshine hours, positively correlates with serum BDNF correlations (Molendijk et al., 2012). Here, BDNF and prospective neurogenesis are modulated by passive environment heat exposure. It was reported by Kojima et al. (2018) that BDNF levels increased by 66% following a 20-min immersion of healthy men in 42°C water compared to another group immersed in 35°C water. Another study by Koyama et al. (2018) found that short-term heat exposure promoted hippocampal neurogenesis when rats were exposed daily to a 1-h heat treatment of 36°C during a 7-day experimental period. Whether physical activity, sun exposure, or hot water exposure, there is a positive correlation between whole-body energy status, adult hippocampal neurogenesis, and neuron survival (Landry and Huang, 2021).

Regarding the amygdala, no evidence exists; however, Kanai et al. (2022) showed in their study that hot and cold environments (8°C and 38°C, respectively) activate the paraventricular thalamic nucleus neurons projecting to brain regions, including the central nucleus of the amygdala, suggesting an involvement in avoidance behaviors or behavioral thermoregulation. Clewett et al. (2013) scanned healthy men’s brains after completing either a stressful cold pressor task (immersing their hands in cold water) or a control task. They discovered that the cold pressor task caused a decrease in how well the amygdala connected with areas of the brain responsible for emotional regulation, such as the orbitofrontal cortex and ventromedial prefrontal cortex. Moreover, the study found that the left amygdala showed more substantial connections with another brain region called the dorsal anterior cingulate cortex after the cold pressor task. This region is known to help reduce the amygdala’s response to stress. However, we suggest that amygdala reactivity to temperature varies depending on emotional states. The effect of short-term, long-term, and intermittent exposure is still unclear.

#### 2.2.5 Air

Recent studies have investigated the potential effects of air pollution on brain structure, particularly in the prefrontal cortex. Gale et al. (2020) conducted a cross-sectional study using data from the UK Biobank to assess the associations between air pollution and gray matter volume in the prefrontal cortex. The study found inverse associations between exposure to particulate matter (PM2.5 and PM10) and nitrogen oxides (NOx) and prefrontal volume after adjusting for potential confounders such as age, sex, education, socioeconomic status, and lifestyle factors. Interestingly, education appeared to have a protective effect against the impact of air pollution on prefrontal volume. These findings suggest that even relatively low levels of air pollution may be associated with reduced prefrontal cortical volume in adults. Ko et al. (2024) investigated the effects of long-term exposure to air pollution on cortical thickness and subcortical volume in a longitudinal neuroimaging study. The study followed a prospective cohort of 361 adults residing in four cities in South Korea over three years. The authors found that a 10-μg/m3 increase in PM10 was associated with reduced whole-brain mean cortical thickness and decreased thickness in the frontal and temporal regions. Similarly, a 10-ppb increase in NO2 was associated with a decline in whole-brain mean cortical thickness and reduced thickness in the frontal, parietal, and temporal regions. The study also found associations between air pollutants and subcortical structures, such as the thalamus volume. The findings from Walker et al. (2020) complement the results of the studies by Gale et al. (2020) and Ko et al. (2024), which focused on the prefrontal cortex. The authors found that a 2-μg/m3 increase in PM2.5 was associated with a 0.32% smaller total cerebral brain volume and 1.46 higher odds of covert brain infarcts. However, there was no clear pattern of association between PM2.5 and hippocampal volume, white matter hyperintensity volume, or extensive white matter hyperintensity volume. Furthermore, a study by Smith et al. (2024) provides valuable insights into the effects of everyday home radon exposure on structural brain morphology in youths. The findings suggest that radon exposure may alter sensitive brain development, impacting gray and white matter trajectories.

The study by Pavlov et al. (2016) also investigated the relationship between wind speed and cerebral cortex activity in women. The researchers found that a decrease in wind speed was associated with a decrease in the activity rate of the right occipital lobe, which is part of the cerebral cortex.

This article hypothesizes that dynamism can be an effective variable for the hippocampus because place cells fire and remap in response to environmental variability fascilitated through multisensory stimuli inducing attentiveness or stimulation and breaks habituation. The stability of place cell firing patterns is influenced by the attention paid to the environment (Kentros et al., 2004).

The abundance of air, facilitated through controlled breathing exercises, can significantly contribute to the amygdala’s role in emotional regulation and neuroplasticity. According to Yuliana (2021), breathing exercises during the COVID-19 pandemic have shown the potential to alter brain structures, including the amygdala, by promoting mindfulness and reducing stress. Additionally, research on meditation-induced neuroplastic changes indicates that such practices can modulate amygdala activity during negative affective processing, further supporting the restorative and plastic potential of the amygdala (Leung et al., 2018). These combined insights underscore the beneficial impact of breathing exercises on the amygdala, enhancing emotional stability and cognitive function through improved neuroplasticity. This potential of breathing exercises offers hope and optimism for the future of brain health.

Hence, we see air as a multifaceted antidote for the enclosed nature of built environments where the air quality, change, and abundance have effects on the cortex, hippocampus, and amygdala, respectively, but that does not disprove that pollution, for instance, may have adverse effects on the latter two brain structures. This section culminates in challenges for the built environment to redefine the presence of air in the indoor space.

#### 2.2.6 Materials

Concrete-dominated environments are often associated with urban settings, which have been linked to increased stress and mental health issues. Lederbogen et al. (2011) found that urban living was associated with more significant amygdala activity in response to stress. While this study does not isolate concrete as a variable, it suggests that the overall urban environment, which heavily utilizes concrete, can affect brain function. A recent study has further explained this. Harris et al. (2023) revealed that gray space was associated with enhanced connectivity between the left amygdala and the default mode network (DMN), which are circuits involved in affective processing, emotion regulation, and psychopathology. Consequently, gray space might be linked to connectivity alterations that could increase the risk of emotion dysregulation. Those studies suggest that gray space, a common feature of built environments, negatively affects the amygdala, unlike natural or green spaces.

Therefore, we strongly advocate for future research to delve into the effects of natural building materials on amygdala activity and long-term neuroplasticity. While the specific influence of gray materials or the absence of nature on the amygdala is yet to be fully understood, we do have evidence that materials like wood have restorative properties (Fell, 2010). This suggests a crucial need for further exploration. More research is needed to fully understand the impact of different material types on the cortex, hippocampus, and amygdala, underscoring the urgency and importance of this topic.

### 2.3 Neurosustainability through aesthetic enrichment

**Figure 5:**
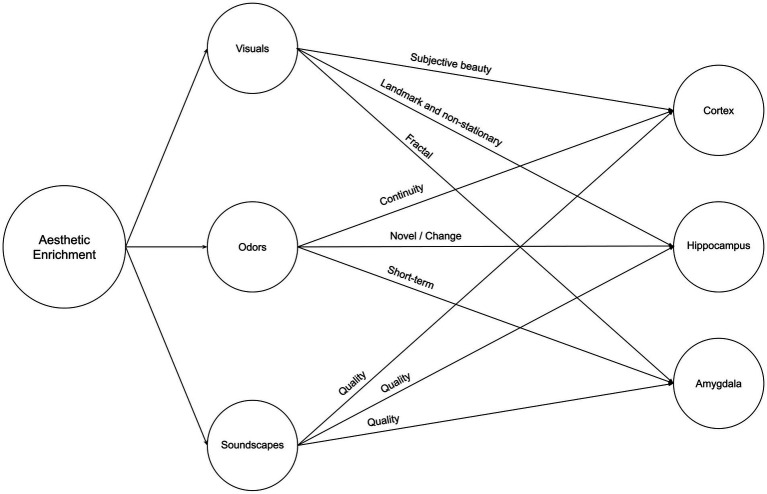
Neurosustainability through aesthetic enrichment: variables and attributes.

Our environment’s aesthetic qualities, encompassing objective and subjective attributes, play a significant role in shaping our cognitive and emotional experiences. As we delve deeper into the relationship between aesthetics and the brain, it becomes increasingly clear that the impact of these sensory variables on neuroplasticity is a topic of great importance and novelty. The aesthetic triad model, as introduced by Chatterjee and Vartanian (2014), highlights the subjective nature of aesthetic experiences and how they relate to an individual’s sense of self and cultural identity. By incorporating this notion into the study of neuroplasticity, we can begin to unravel the complex interplay between subjective aesthetic experiences and the brain’s adaptive responses. Moreover, the novelty of this article lies in its exploration of how aesthetic variables, such as visual, olfactory, and auditory cues, can influence neuroplasticity in the cortex, hippocampus, and amygdala – three brain regions closely associated with perception, memory, and emotion. This topic becomes of equal importance to spatial and natural enrichment because aesthetic enrichment is present in higher variability as we see, for instance, different interior styles, each consisting of various color schemes, complexity of items, and difference in scale. Therefore, while very little research has been conducted on the impact of aesthetics on neuroplasticity, we cannot ignore the prospective impact of those variables on neuroplasticity. Figure 5 illustrates a summary of Neurosustainability through aesthetic enrichment before each variable and attribute is expounded.

#### 2.3.1 Visuals

Visual aesthetics are complex, consisting of various elements such as color, scale, proximity, and so forth. Each of those variables is even more variable within each scene. This section elaborates on how visual aspects of aesthetic experiences can impact neuroplasticity processes.

Purely aesthetic experiences of natural landscapes and interior environments, regardless of the visual element, impacted cortical plasticity. For instance, higher subjective neighborhood beauty was associated with larger bilateral medial orbitofrontal cortex and insula volumes and, interestingly, was not associated with objective measures such as green space, blue space, or plant diversity (Tani et al., 2022). Furthermore, subjective home environment qualities, such as crowdedness or perceived hazard, are associated with thinner prefrontal cortices regardless of socioeconomic status or psychosocial factors (Uy et al., 2019).

Regarding the hippocampus, McCormick et al. (2021) discovered that the hippocampus responded more to scene stimuli, with color detection engaging the posterior hippocampus and layout detection engaging the anterior hippocampus, while scene complexity did not influence hippocampal activity. The hippocampus exhibits selectivity for specific sensory feature combinations, even during novel experiences (Priestley et al., 2022). More research is needed on that part, with prospective rodent-related literature providing the baseline for that part of the research. For instance, the hippocampus processes visual cues as stable landmarks for orientation or navigation or as non-stationary objects or features in the environment used for associative learning (Scaplen et al., 2014).

Little research is available about the amygdala, but one study supports that it might be sensitive to natural aesthetics. Viewing a typical flower image led to decreased activation in the right amygdala-hippocampus region, unlike viewing a mosaic flower (Mochizuki-Kawai et al., 2020).

#### 2.3.2 Odors

Olfaction plays various roles in the cortex, hippocampus, and amygdala. Firstly, olfaction was reported by Kokubun et al. (2024) as effective, where they found that continuous inhalation of essential oil increases gray matter volume. Secondly, in contrast with the visual cue, olfactory cues, using flower scents, helped participants in the stress condition to have reduced brain activation in the right amygdala in the short term (Ai et al., 2023). However, the amygdala did not change after one month in another study (Kokubun et al., 2024). Thirdly, for the hippocampus, Kulvicius et al. (2008) emphasize the additive effect of olfactory cues with navigation, supporting place cell firing in the hippocampus but in rodents. More research is needed on humans. Besides, Zhang and Manahan-Vaughan (2015) suggested that place cells in the hippocampus also occur when olfactory and auditory cues are effective. Olfaction, therefore, is multifaceted and can have additive effects on the aesthetic experience.

#### 2.3.3 Soundscapes

Most research suggests that the quality of soundscapes matters most for the cortex, hippocampus, and amygdala. For instance, excessive noise was found to be associated with thinner prefrontal cortices by Uy et al. (2019), which is confirmed by another study by Simon et al. (2022), who tested whether high everyday noise exposure among children affected the cortex, finding that excessive levels of noise reduced cortical thickness in the left inferior frontal gyrus, associated with differences in language processing differences. On the other hand, while there is no direct evidence on auditory aesthetics and hippocampus or amygdala plasticity, we have indirect evidence that the latter may be improved by natural soundscapes than urban or artificial sound or noise (Gould van Praag et al., 2017; Aletta et al., 2018; Hahad et al., 2019). Stobbe et al. (2023) investigated the effects of listening to natural and urban soundscapes on cognitive performance, mood, stress reactivity, and brain activity during a cognitive task. The authors found that exposure to natural soundscapes, compared to urban soundscapes, decreased negative affect and higher aesthetic preference ratings. Although the effect did not reach statistical significance, listening to natural soundscapes also led to better cognitive performance.

Regarding brain activity, the study found an exploratory result suggesting that listening to urban soundscapes was associated with increased activity in the superior temporal gyrus (STG) during a subsequent cognitive task (dual n-back). The STG is involved in auditory processing and language comprehension. The authors speculate that this increased STG activity could indicate less efficient information processing in early primary sensory areas due to exposure to urban soundscapes. However, it is essential to note that this result was not statistically corrected and should be interpreted cautiously. Last, Zhang et al. (2021) demonstrated that moderate noise levels (∼65 dB SPL), which do not significantly affect stress levels or peripheral hearing, can still substantially impair hippocampus-dependent learning and memory by altering synaptic plasticity in a rodent model. The authors suggest that the negative impacts of chronic exposure to moderate noise may be underestimated and emphasize the need for further research to better understand the potential hazards of noise pollution in modern human environments.

### 2.4 Neurosustainability through social enrichment

Social enrichment, including neighborhood deprivation and loneliness, significantly impacts brain morphology and function. Understanding these influences is crucial for promoting Neurosustainability. However, the focus of this article on social enrichment aims to prevent the negative impacts associated with housing, for instance, in the case of sole living. In this part, we present co-living as a neighborhood-level social enrichment, while loneliness is a house-level form of social deprivation. On the one hand, living in deprived neighborhoods (small area-level social disadvantage) throughout the life course has been associated with adverse brain morphologies. Specifically, these environments correlate with reduced total brain and gray matter volume, thinner cortex, and lower general fractional anisotropy (Baranyi et al., 2023a,b). Fractional anisotropy is a valuable measure of the microstructural integrity and organization of white matter in the brain. It provides insights into both normal development and various neurological and psychiatric disorders, highlighting the profound impact of social disadvantage on brain health.

On the other hand, social isolation has also been linked to significant changes in brain structure. Lammer et al. (2023) found that social isolation is associated with reduced cortical thickness and hippocampal volume clusters. Last but not least, it was reported that acute social isolation and regrouping cause short—and long-term molecular changes in the medial amygdala of rat subjects, which supports that the COVID-19 pandemic lockdown has had adverse effects that may be pathological (Lavenda-Grosberg et al., 2022).

These findings underscore the critical role of social interactions in maintaining brain health. They also suggest that social isolation can lead to structural brain changes, potentially predisposing individuals to cognitive decline and mental health disorders. This highlights the urgent need to address social isolation, as it can have significant negative effects on brain health.

## 3 Neurosustainability: Changes, challenges, chances

### 3.1 Changes

Change represents a shift in perspective across disciplines that walk in parallel, encouraging each of them to expand their perspective in order to reduce the gaps that lead to the deterioration of the neuroplastic human brain.

#### 3.1.1 Neurosustainability and neuroarchitecture: architecture for the brain and architecture of the brain

First and foremost, we aim to expound on the distinction between Neurosustainability and Neuroarchitecture, why the latter receives criticisms, and why the former may be a better literal fit. Neuroarchitecture combines two primary fields: neuroscience, specifically, which studies the nervous system, and architecture, which focuses on the design and construction of buildings. Neuroarchitecture suggests an intersection where the built environment influences and is influenced by human neural activity. However, the interpretation of this term can vary significantly across disciplines; hence, it receives criticism.

In architecture, Neuroarchitecture is often viewed through the lens of how architectural design can impact human well-being, cognitive function, and emotional state. Its key concepts mainly focus on spatial cognition but expand beyond this scope. Neuroscientists may interpret Neuroarchitecture as a study of how the brain processes and responds to architectural spaces, and sometimes worse for architects, neuroscientists interpret Neuroarchitecture as merely the architecture of the brain itself, such as structure, neurons (Helfrich-Förster, 2003; Wolff et al., 2015). While Neuroarchitecture is indeed literally translated as the architecture of the brain, Neurosustainability refers to the process of sustainability of the human brain’s neuroplasticity processes, which is something that needs to take place through sustainability practices that are already renowned for focusing on building and the environment. Furthermore, the environment is the brain’s architect, and that has been for thousands of years.

Neurosustainability can be a better representative for interdisciplinary research between neuroscience and architecture to continuously improve the architecture of the brain since it is an ongoing process and does not happen once. However, both Neuroarchitecture and Neurosustainability can keep walking parallel roads and often meet at certain points.

#### 3.1.2 Neurosustainability and sustainability: plasticity before and through the planet

As introduced earlier in the previous section, sustainability practices aim to save the planet, the environment, and energy consumption. However, despite the interest in improving health and well-being at specific points, neuroplasticity and the ongoing effect on the human brain require sustainable research and practice attention. Neurosustainability, a novel field that examines the intersection of neuroplasticity and sustainability, underscores the importance of incorporating human cognitive and neural processes into sustainability efforts.

Sustainability practices in the built environment primarily focus on reducing environmental impact through various strategies. These strategies include implementing energy-efficient systems and technologies to reduce energy consumption and greenhouse gas emissions (Wu et al., 2016; Gassar and Cha, 2020; Pham et al., 2020; Skillington et al., 2022), using sustainable materials and promoting recycling and the reuse of resources to minimize waste (He et al., 2020; Soni et al., 2022), designing systems that reduce water usage and promote water recycling, and adopting certifications such as LEED (Leadership in Energy and Environmental Design) to ensure buildings meet specific environmental criteria (Awadh, 2017; Ferreira et al., 2023; Wijesooriya et al., 2023). Additionally, there is an emphasis on enhancing indoor air quality, natural lighting, and thermal comfort to improve the health and well-being of occupants (Elnaklah et al., 2021; Mannan and Al-Ghamdi, 2021). While these practices are crucial for mitigating environmental impact and promoting the health of occupants, they do not consider the built environment’s profound effects on the human brain, particularly in terms of neuroplasticity^1^.

In order to design sustainable environments, it is essential to consider how the built environment impacts the brain’s plasticity and sustains neurons over time. This integration can lead to more holistic and effective sustainability practices, which are argued to be an integral part of the architectural decision-making process (Feria and Amado, 2019). Artificial intelligence can also aid this process (Nishant et al., 2020; Singh et al., 2020; Merabet et al., 2021; Bibri et al., 2024).

#### 3.1.3 Neurosustainability and neuroaesthetics: what the brain wants and what the brain needs

The distinction between Neuroaesthetics and Neurosustainability is as simple as the difference between what the brain wants (Derke et al., 2023) and what it needs. Neuroaesthetics, a field that studies the neural basis of aesthetic experiences, explores how the brain processes beauty and art. It focuses on what the brain finds pleasurable and satisfying, delving into the sensory and emotional responses elicited by aesthetic stimuli (Chatterjee and Vartanian, 2014). Neurosustainability, however novel, complements rather than positions itself as an opposite terminology.

Neuroesthetics investigates how different forms of art, architecture, and natural beauty stimulate the brain (Pearce et al., 2016; Coburn et al., 2017; Chatterjee et al., 2021). It examines how visual, auditory, and tactile experiences evoke pleasure, calmness, or excitement. For instance, seeing a well-designed building, a beautiful painting, or a harmonious landscape can trigger positive emotional responses and activate reward pathways in the brain. These aesthetic experiences are essential because they enhance mood, reduce stress, and promote well-being. However, while these “wants” of the brain are crucial for immediate satisfaction and happiness, they do not necessarily address the deeper “needs” required for sustainable neuroplasticity processes.

Neurosustainability goes beyond immediate gratification to focus on the essential needs of the brain for long-term brain health and resilience through a dynamic relationship with the environment. Aesthetic enrichment is part of this novel Neurosustainability theory and framework, which guides how to orchestrate the human brain through spatial, natural, aesthetic, and social enrichment that often takes place simultaneously.

#### 3.1.4 Neurosustainability and epigenetics: bridging the gap between science and design

While epigenetics offers valuable insights into how environmental factors influence brain health and plasticity, translating this knowledge into practical design strategies for built environments presents significant challenges. One of the main obstacles is the complex and multifaceted nature of epigenetic mechanisms, which can be challenging to interpret and consider during architectural design.

Another challenge lies in the long-term and transgenerational effects of epigenetic modifications. While the Neurosustainability framework emphasizes the importance of creating environments that support brain health over the lifespan, the epigenetic impact of environmental exposures may take time to become apparent. Designers ought to consider the potential long-term consequences of their design choices and develop strategies to mitigate the risk of adverse epigenetic effects over time.

It is essential to foster interdisciplinary collaboration between neuroscientists, epigeneticists, and design professionals. By working together, these experts can develop a shared understanding of the complex relationship between the environment, epigenetics, and brain health. This collaborative approach can help bridge the gap between scientific knowledge and practical design applications, enabling the creation of environments that effectively promote brain health and sustainable plasticity.

### 3.2 Challenges

Urban spaces face many challenges in fully integrating the variables and attributes that promote neuroplasticity. One major challenge is increasing navigational complexity, which is crucial given the cognitive benefits of navigational demands, such as those of taxi drivers. While urban design traditionally focuses on vehicular routes, creating more interconnected pedestrian and cycling paths would require reimagining infrastructure with walking and cycling, for instance, in mind. With strategic placement of landmarks, including public art and green spaces, navigating complex urban realms could become cognitively stimulating while encouraging physical activity.

Contemporary architectural design may pose real challenges to Neurosustainability. High-rise buildings present one of the most significant challenges to maximizing sky views in urban spaces. As density increases vertically to accommodate growing populations, tall towers inevitably block out the natural sky when in close proximity. This is problematic because research has shown sky visibility to be the most impactful predictor of prefrontal cortical plasticity in children compared to just tree cover alone. One way to help overcome the obstruction of sky views in high-density areas is through strategic step-backs in building design.

High-rise buildings pose unique challenges to maintaining natural wind movement patterns within dense urban contexts. The towering vertical typology common to many city skylines disrupts ambient airflow dynamics at the pedestrian level when structures are placed too closely together. Unbroken concrete and glass walls inhibit cross breezes that traditionally circulated through low-height buildings. Without sufficient spacing between towers, updrafts and downdrafts that carry warm and cool air masses lose their fluid exchange, resulting in static pockets of stagnant air at street level.

Residential greenness faces challenges in overcoming urban canyon effects and the prevalence of gray infrastructure; however, strategic planting provides opportunities. While green walls and roofs expand canopy coverage, research indicates natural trees uniquely impact the amygdala versus artificial structures alone. Trees offer sensory complexity through sights, scents, and textures that engineered systems cannot replicate. Their living biomaterial sequesters carbon in a closed-loop process versus resource-intensive technical solutions. Other seemingly innovative solutions, such as digital or liquid trees, however, miss out on an integral part of the presence of trees, and future innovative solutions should aim at bringing natural trees into urban spaces again rather than providing a replica, which again transforms the relationship between nature and humans from top-down to bottom-up and thus negatively affecting the human brain.

Cities with high spatial complexity from extensive street networks, diverse layouts, and abundant commercial spaces provide an enriched environment beneficial for hippocampal neuroplasticity. The extensive navigational cues and opportunities for exploration effectively activate mechanisms like adult neurogenesis through hippocampal mapping. However, such vibrant urban centers often remain lively late into the night. While the prolonged activity stimulates cognitive mapping abilities by extending the period of environmental engagement, it also exposes residents to nighttime light that is disruptive to circadian rhythms and critical for hippocampal regeneration. Without sufficient dark periods aligned with natural sleeping cycles, overstimulation risks compromising the plasticity it aims to improve.

In contrast, cities with more straightforward navigational structures, uniform layouts, and sparse commercial options offer fewer spatial stimuli during peak activity hours. This deprives the hippocampus of diverse cues needed to optimize cognitive representations. Also, earlier closures minimize circadian conflicts by restoring circadian alignment in sleep. As densities increase and commercial hours dwindle earlier, dwellers face confined quarters posing analogous deprivation to impoverished cities. Without environmental modification embracing navigational puzzles stimulating mapping circuits from within, indoor realms represent a more deprived miniature of the urban environment.

Built environments commonly consist of straightforward layouts with minimal interior navigation like direct corridors. Straightforward interior navigation undermine hippocampal neurogenesis ability and cortical thickening. Building designs can experiment spatial puzzles by including more corridors, staircases, complex layouts, and multi-floor circulation routes. This increases navigational demands within living spaces, layout complexity, and more. By embedding complexity that necessitates more walking intercourse between diversified indoor territories, built environment layouts can effectively promote higher levels of physical activity achieved effortlessly as a natural consequence of inhabiting neuroplasticity-enriched living spaces.

Incorporating more natural elements indoors poses immense potential to enrich neuroplasticity. Prioritizing clerestories and skylights over conventional windows could better introduce sky exposures, which have been shown to impact regions like the prefrontal cortex uniquely compared to mere tree views. However, it will challenge the architectural design where multi-story buildings are divided into apartments for different owners. Furthermore, density targets for indoor greenery must also be established, as biophilic stimuli hold additive effects when experienced residentially versus solely outdoors. Thoughtful plant placements providing sensations across fractal visual, olfactory, and tactile domains may stimulate structures like the amygdala. Introducing biophilic soundscapes could further spatially activate functions such as emotional processing. Optimized ventilation circulation and emissions filtration through foliage could aid indoor air purification, which has been proven critical to the cerebral cortex. Considering lighting intricacies like natural circadian alignment, lighting quality, intensity, and duration variations impacting the amygdala, as well as thermal implications on neurogenesis versus the cortex, will be indispensable for architectural Neurosustainability. Tackling these complex natural complexity challenges can help spatially nourish cognitive longevity through dwellings better attuned to the brain’s intrinsic connectivity with nature.

Integrating dynamic aesthetic complexity into indoor environments holds immense potential for enriching neuroplasticity. Interior styles intrinsically offer temporally fluctuating amalgamations of diverse sensory stimuli by incorporating adjustable visual components. Varying schemes across color palettes, geometric compositions, scaled motifs, and material textures may introduce intricate combinatorial sensory node networks primed to stimulate hippocampal mapping circuits through their recombination across evolving layouts. Regular rearrangement of interior variables may hold the potential for stimulating hippocampal neurogenesis. However, it is yet to be known which interior variables are most effective and the frequency of change.

### 3.3 Chances

#### 3.3.1 Future research

The paper introduces the novel concept of Neurosustainability and offers a framework for interdisciplinary research bridging neuroplasticity, design, and sustainability. Firstly, there is an opportunity to conduct comparative studies of urban neighborhoods and building designs that vary specifically in their degree of spatial complexity. Metrics like the number of intersections, dead-ends, and floor plan variations could be quantified. This research could inform evidence-based spatial guidelines for optimizing layouts of residential buildings, workspaces and other building types. Secondly, interdisciplinary research combining longitudinal cohort studies or experiments could investigate variations in cortical, hippocampal, and amygdalar responses to natural complexities indoors from childhood through aging. This may provide insights into optimizing environmental interventions tailored to critical neurodevelopmental periods or neurodegenerative prevention. Thirdly, interdisciplinary research could involve randomized controlled trials varying apartment aesthetics on a set schedule, where the interior elements changed are not merely decorative but include items intended to provide cognitive stimulation. Lastly, an opportunity lies in cross-cultural studies exploring how different cultural constructs of social relationships, individualism/collectivism, influence neuroplasticity and mental health outcomes. This interdisciplinary work may help optimize housing/neighborhood designs respecting diverse social complexity needs.

#### 3.3.2 Interrelated variables

While several variables are identified within each enrichment type, the framework does not imply that these variables or attributes cannot also relate or interact with one another. This provides a significant opportunity for interdisciplinary research to study Neurosustainability more holistically as an integrative concept, with the flexibility to formulate hypotheses exploring combinations and synergies between variables across domains. For example, navigation under spatial complexity could be investigated jointly with physical activity attributes or sky views in natural complexity combined with various visual elements in aesthetic enrichment. Lighting as a natural enrichment factor may also correlate with perceptual responses to visuals. Interactions between temperature and physical activity through architectural design can be two facets of whole-body energy and metabolism. This interdisciplinary approach embraces the interactive nature through which real-world environments influence the brain, avoiding the examination of variables in isolation. It also helps establish Neurosustainability optimally nourished through coordinated multi-factorial lifestyle and design interventions.

#### 3.3.3 Methodologies

While directly assessing neuroplasticity processes like neurogenesis require invasive neurological methods challenging to apply in human studies, the paper discusses potential indicators like, BDNF/NGF levels, and neuroimaging metrics that provide opportunities for non-invasive research. Measures of cortical thickness, white/gray matter integrity from MRI, and hippocampal volume changes can help evaluate plasticity responses to environmental interventions. Amygdala plasticity could also be traced using MRI or EEG to detect changes from short or long-term exposures. Psychological metrics are also discussed, like assessments of anxiety, depression, stress, and cognition, which may relate to plasticity in regions governing those functions. Subjective measures of stress or restorativeness could also provide indicators for amygdala responses. This presents an opportunity for future interdisciplinary Neurosustainability research that is not limited to solely invasive means. Large-scale cohort studies combining various methodologies discussed would help establish relationships between lifestyle/design factors and neural resilience markers.

#### 3.3.4 Individual differences

Interdisciplinary Neurosustainability research should account for potential influences of gender and age that may impact study outcomes. As noted throughout the paper, several studies report women exhibiting more significant relationships between environmental exposures and neural responses compared to men. Age is another factor, as specific neuroplasticity processes like neurogenesis have generally been found to decline with older age. Careful study design controlling for these potential covariates will be necessary. Strategically focusing recruitment to compare gender-matched or age-defined groups could help isolate differentiated effects. Accounting for individual variability will strengthen the understanding of how lifestyle and design interventions may differently influence neuroplasticity across populations over the lifespan. This will better support cognitive longevity through gender-sensitive and age-appropriate Neurosustainability practices.

## 4 Conclusion

This article introduced the novel theory and framework of Neurosustainability to foster crucial conversations between disciplines regarding the built environment’s profound influence on the human brain’s continuous neuroplasticity. By reviewing spatial, natural, aesthetic and social enrichment means through a neuroplasticity lens, we illuminated how nature’s multifaceted richness has sculpted the ever-evolving human brain across millennia. Yet modern constraints increasingly divorce our environments from nature’s quintessential essence, with drastic consequences for neuroplasticity.

As sedentary living, isolation and high stress compromise neuroplasticity, issues like depression, dementia and declining well-being are downstream effects of poor neuroplasticity support. This status quo cannot persist if we seek to architect environments supporting lifelong sustainable neuroplasticity. Neurosustainability challenges us to transcend contemporary thinking by architecting environments that in turn become good architects for our brains, optimizing neuroplasticity across generations to come.

Achieving this will require urgent interdisciplinary action. Designers, researchers and policymakers should prioritize neuroplastic sustainability through deliberate coordination of enriched variables attuned to sculpting key brain regions. Only by restoring nature’s complexity into daily living can we stem avoidable neural decline and safeguard the sustainability and evolution of our brains.

The concept emphasizes the importance of environmental enrichment through spatial, natural, aesthetic, and social complexities. These complexities, deeply embedded in nature, are crucial for sustaining and enhancing the brain’s neuroplastic processes. Incorporating elements of nature and complexity into modern living spaces can mitigate the adverse effects of contemporary lifestyles, such as cognitive decline, mental health disorders, and social isolation.

Ultimately, Neurosustainability calls for a reconceptualization of our interaction with the environment. It encourages a move beyond the static, limited, predictable, and often detrimental aspects of contemporary living toward a dynamic and enriching coexistence with nature. Through this innovative approach, we can harness the quintessential essence of nature to promote cognitive health and psychological well-being, ensuring that the human brain continues to thrive in an ever-changing world.

## Data Availability

The original contributions presented in the study are included in the article/supplementary material, further inquiries can be directed to the corresponding author.

## Footnotes

^1^This paper has introduced a novel theory titled "Neurosustainability," which explores the role of the physical environment—both natural and built—in sustaining neuroplasticity through environmental enrichment strategies. Even though we raise awareness among researchers in sustainability, the word sustainability used in this article refers to the quintessential meaning of the word "sustainability" beyond its applied definition. It has come to the author’s attention that the term Neurosustainability was previously utilized in a different context by Özer (2021). This prior usage was not identified during this article’s ideation phase or literature review process, as the primary database searches conducted do not include the source containing Özer’s work. Özer’s paper refers to a different meaning of the term Neurosustainability, however, which does not convey the same meaning introduced in this article and does not infringe its originality. However, as this article raised awareness for researchers in sustainability to be Neurosustainabilityconscious, we acknowledge Özer’s work as relevant to the awareness raised. This situation underscores the multidisciplinary interest in and relevance of Neurosustainability and sets a foundation for future scholarly discourse to refine and differentiate terminology usage within varied research contexts. Future research may cite only one of the two articles depending on the discipline; therefore, future research may no longer need to cite both articles unless needed.

## References

[ref1] Ai Y. Hummel T. Nie H. Yang J. Han P. (2023). Reduced neural responses to pleasant odor stimuli after acute psychological stress is associated with cortisol reactivity. NeuroImage 284:120474. doi: 10.1016/j.neuroimage.2023.120474 38008298

[ref2] Aletta F. Oberman T. Kang J. (2018). Associations between positive health-related effects and soundscapes perceptual constructs: a systematic review. Int. J. Environ. Res. Public Health 15:2392. doi: 10.3390/ijerph15112392, PMID: 30380601 PMC6266166

[ref3] Alqassim A. Y. Mahfouz M. S. Hakami M. M. Al Faqih A. A. Shugairi A. A. Alsanosy M. R.. (2022). Depression, anxiety, stress and their association with the use of electronic devices among adolescents during the COVID-19 pandemic. Int. J. Ment. Health Promot. 24, 251–262. doi: 10.32604/ijmhp.2022.019000

[ref4] Awadh O. (2017). Sustainability and green building rating systems: LEED, BREEAM, GSAS and Estidama critical analysis. J. Build. Eng. 11, 25–29. doi: 10.1016/j.jobe.2017.03.010

[ref5] Baceviciene M. Jankauskiene R. (2022). The mediating effect of nature restorativeness, stress level, and nature connectedness in the association between nature exposure and quality of life. Int. J. Environ. Res. Public Health 19:2098. doi: 10.3390/ijerph19042098, PMID: 35206285 PMC8871825

[ref6] Baker N. Steemers K. (2019). Healthy homes: Designing with light and air for sustainability and wellbeing. London: Riba Publishing.

[ref7] Baranyi G. Buchanan C. R. Conole E. L. Backhouse E. V. Maniega S. M. Hernandez M. V.. (2023b). Life-course neighbourhood deprivation and brain structure in older adults: the Lothian birth cohort 1936. medRxiv. doi: 10.1038/s41380-024-02591-9 PMC11541210 38773266

[ref8] Baranyi G. Conte F. Deary I. J. Shortt N. Thompson C. W. Cox S. R.. (2023a). Neighbourhood deprivation across eight decades and late-life cognitive function in the Lothian birth cohort 1936: a life-course study. Age Ageing 52:afad056. doi: 10.1093/ageing/afad056 37097769 PMC10128164

[ref9] Besser L. M. Lovasi G. S. Michael Y. L. Garg P. Hirsch J. A. Siscovick D.. (2021). Associations between neighborhood greenspace and brain imaging measures in non-demented older adults: the cardiovascular health study. Soc. Psychiatry Psychiatr. Epidemiol. 56, 1575–1585. doi: 10.1007/s00127-020-02000-w, PMID: 33388800 PMC8253869

[ref10] Besser L. M. Lovasi G. S. Zambrano J. J. Camacho S. Dhanekula D. Michael Y. L.. (2023). Neighborhood greenspace and neighborhood income associated with white matter grade worsening: cardiovascular health study. Alzheimer's Dement. 15:e12484. doi: 10.1002/dad2.12484 PMC10598801 37885920

[ref11] Bibri S. E. Krogstie J. Kaboli A. Alahi A. (2024). Smarter eco-cities and their leading-edge artificial intelligence of things solutions for environmental sustainability: a comprehensive systematic review. Environ. Sci. Ecotechnol. 19:100330. doi: 10.1016/j.ese.2023.100330, PMID: 38021367 PMC10656232

[ref12] Bird C. M. Burgess N. (2008). The hippocampus and memory: insights from spatial processing. Nat. Rev. Neurosci. 9, 182–194. doi: 10.1038/nrn2335, PMID: 18270514

[ref13] Birkel L. (2017). Decreased use of spatial pattern separation in contemporary lifestyles may contribute to hippocampal atrophy and diminish mental health. Med. Hypotheses 107, 55–63. doi: 10.1016/j.mehy.2017.07.012, PMID: 28915964

[ref14] Brown C. J. Jeon S. Ng Y. T. Lee S. Fingerman K. L. Charles S. T. (2023). Switching it up: activity diversity and cognitive functioning in later life. Psychol. Aging. doi: 10.1037/pag0000770 PMC10528947 37535516

[ref15] Bumgarner J. R. Walker W. H. Quintana D. D. White R. C. Richmond A. A. Meléndez-Fernández O. H.. (2023). Acute exposure to artificial light at night alters hippocampal vascular structure in mice. Iscience 26:106996. doi: 10.1016/j.isci.2023.106996 37534143 PMC10391664

[ref16] Cadenas-Sanchez C. Migueles J. H. Verdejo-Román J. Erickson K. I. Esteban-Cornejo I. Catena A.. (2023). Physical activity, sedentary time, and fitness in relation to brain shapes in children with overweight/obesity: links to intelligence. Scand. J. Med. Sci. Sports 33, 319–330. doi: 10.1111/sms.14263, PMID: 36337011 PMC11227654

[ref17] Campbell I. Balda F. Sharifpour R. Paparella I. Beckers E. Berger A.. (2024). Exposure to light supresses the activity of the medial and superior amygdala during emotional processing. bioRxiv. doi: 10.1101/2024.04.25.591085

[ref18] Cavanna A. E. Trimble M. R. (2006). The precuneus: a review of its functional anatomy and behavioural correlates. Brain 129, 564–583. doi: 10.1093/brain/awl004 16399806

[ref19] Cenkner D. P. Burgess H. J. Huizenga B. Duval E. R. Kim H. M. Phan K. L.. (2022). Morning light treatment for traumatic stress: the role of amygdala reactivity study protocol. PLoS One 17:e0269502. doi: 10.1371/journal.pone.0269502, PMID: 35675275 PMC9176814

[ref20] Champagne F. A. (2010). Epigenetic influence of social experiences across the lifespan. Dev. Psychobiol. 52, 299–311. doi: 10.1002/dev.20436 20175106

[ref21] Chatterjee A. Coburn A. Weinberger A. (2021). The neuroaesthetics of architectural spaces. Cogn. Process. 22, 115–120. doi: 10.1007/s10339-021-01043-4, PMID: 34448969

[ref22] Chatterjee A. Vartanian O. (2014). Neuroaesthetics. Trends Cogn. Sci. 18, 370–375. doi: 10.1016/j.tics.2014.03.003 24768244

[ref23] Chen Y. C. Chen C. Martínez R. M. Etnier J. L. Cheng Y. (2019). Habitual physical activity mediates the acute exercise-induced modulation of anxiety-related amygdala functional connectivity. Sci. Rep. 9:19787. doi: 10.1038/s41598-019-56226-z, PMID: 31875047 PMC6930267

[ref24] Clemenson G. D. Deng W. Gage F. H. (2015). Environmental enrichment and neurogenesis: from mice to humans. Curr. Opin. Behav. Sci. 4, 56–62. doi: 10.1016/j.cobeha.2015.02.005

[ref25] Clewett D. Schoeke A. Mather M. (2013). Amygdala functional connectivity is reduced after the cold pressor task. Cogn. Affect. Behav. Neurosci. 13, 501–518. doi: 10.3758/s13415-013-0162-x, PMID: 23645370 PMC3778131

[ref26] Clifford A. Lang L. Chen R. Anstey K. J. Seaton A. (2016). Exposure to air pollution and cognitive functioning across the life course–a systematic literature review. Environ. Res. 147, 383–398. doi: 10.1016/j.envres.2016.01.018, PMID: 26945620

[ref27] Coburn A. Vartanian O. Chatterjee A. (2017). Buildings, beauty, and the brain: a neuroscience of architectural experience. J. Cogn. Neurosci. 29, 1521–1531. doi: 10.1162/jocn_a_01146, PMID: 28493809

[ref28] Coburn A. Vartanian O. Kenett Y. N. Nadal M. Hartung F. Hayn-Leichsenring G.. (2020). Psychological and neural responses to architectural interiors. Cortex 126, 217–241. doi: 10.1016/j.cortex.2020.01.009, PMID: 32092492

[ref29] Costa G. A. Silva N. K. D. G. T. Marianno P. Chivers P. Bailey A. Camarini R. (2023). Environmental enrichment increased Bdnf transcripts in the prefrontal cortex: implications for an epigenetically controlled mechanism. Neuroscience 526, 277–289. doi: 10.1016/j.neuroscience.2023.07.001 37419403

[ref30] Dadvand P. Pujol J. Macià D. Martínez-Vilavella G. Blanco-Hinojo L. Mortamais M.. (2018). The association between lifelong greenspace exposure and 3-dimensional brain magnetic resonance imaging in Barcelona schoolchildren. Environ. Health Perspect. 126:027012. doi: 10.1289/EHP1876, PMID: 29504939 PMC6066357

[ref31] Deantoni M. Villemonteix T. Balteau E. Schmidt C. Peigneux P. (2021). Post-training sleep modulates topographical relearning-dependent resting state activity. Brain Sci. 11:476. doi: 10.3390/brainsci11040476 33918574 PMC8069225

[ref32] Derke F. Filipović-Grčić L. Raguž M. Lasić S. Orešković D. Demarin V. (2023). “Neuroaesthetics: how we like what we like” in Mind, brain and education (Cham: Springer International Publishing), 1–12.

[ref33] Di Liegro C. M. Schiera G. Proia P. Di Liegro I. (2019). Physical activity and brain health. Genes 10:720. doi: 10.3390/genes10090720, PMID: 31533339 PMC6770965

[ref34] Diamond M. C. Krech D. Rosenzweig M. R. (1964). The effects of an enriched environment on the histology of the rat cerebral cortex. J. Comp. Neurol. 123, 111–119. doi: 10.1002/cne.901230110 14199261

[ref35] Domingos C. Pêgo J. M. Santos N. C. (2021b). Effects of physical activity on brain function and structure in older adults: a systematic review. Behav. Brain Res. 402:113061. doi: 10.1016/j.bbr.2020.113061, PMID: 33359570

[ref36] Domingos C. Picó-Pérez M. Magalhães R. Moreira M. Sousa N. Pêgo J. M.. (2021a). Free-living physical activity measured with a wearable device is associated with larger hippocampus volume and greater functional connectivity in healthy older adults: An observational, cross-sectional study in northern Portugal. Front. Aging Neurosci. 13:729060. doi: 10.3389/fnagi.2021.729060, PMID: 34916921 PMC8670087

[ref37] Elnaklah R. Walker I. Natarajan S. (2021). Moving to a green building: indoor environment quality, thermal comfort and health. Build. Environ. 191:107592. doi: 10.1016/j.buildenv.2021.107592

[ref38] Falck R. S. Hsu C. L. Best J. R. Li L. C. Egbert A. R. Liu-Ambrose T. (2020). Not just for joints: the associations of moderate-to-vigorous physical activity and sedentary behavior with brain cortical thickness. Med. Sci. Sports Exerc. 52, 2217–2223. doi: 10.1249/MSS.0000000000002374, PMID: 32936595

[ref39] Fares R. P. Belmeguenai A. Sanchez P. E. Kouchi H. Y. Bodennec J. Morales A.. (2013). Standardized environmental enrichment supports enhanced brain plasticity in healthy rats and prevents cognitive impairment in epileptic rats. PLoS One 8:e53888. doi: 10.1371/journal.pone.0053888, PMID: 23342033 PMC3544705

[ref40] Fell D. R. (2010). Wood in the human environment: Restorative properties of wood in the built indoor environment. Doctoral dissertation. Vancouver: University of British Columbia.

[ref41] Fenton A. A. (2024). Remapping revisited: how the hippocampus represents different spaces. Nat. Rev. Neurosci. 25, 428–448. doi: 10.1038/s41583-024-00817-x 38714834

[ref42] Feria M. Amado M. (2019). Architectural design: sustainability in the decision-making process. Buildings 9:135. doi: 10.3390/buildings9050135

[ref43] Ferreira A. Pinheiro M. D. de Brito J. Mateus R. (2023). A critical analysis of LEED, BREEAM and DGNB as sustainability assessment methods for retail buildings. J. Build. Eng. 66:105825. doi: 10.1016/j.jobe.2023.105825

[ref44] Firth J. Stubbs B. Vancampfort D. Schuch F. Lagopoulos J. Rosenbaum S.. (2018). Effect of aerobic exercise on hippocampal volume in humans: a systematic review and meta-analysis. NeuroImage 166, 230–238. doi: 10.1016/j.neuroimage.2017.11.007, PMID: 29113943

[ref45] Fischer A. Sananbenesi F. Wang X. Dobbin M. Tsai L. H. (2007). Recovery of learning and memory is associated with chromatin remodelling. Nature 447, 178–182. doi: 10.1038/nature05772, PMID: 17468743

[ref46] Fisher P. M. Madsen M. K. Mc Mahon B. Holst K. K. Andersen S. B. Laursen H. R.. (2014). Three-week bright-light intervention has dose-related effects on threat-related corticolimbic reactivity and functional coupling. Biol. Psychiatry 76, 332–339. doi: 10.1016/j.biopsych.2013.11.031, PMID: 24439303

[ref47] Folville A. Willems S. Cheriet N. Geurten M. Guillemin C. Muto V.. (2023). Well-being during COVID-19-related first lockdown: relationship with autobiographical memory and experiential diversity. Appl. Cogn. Psychol. 37, 1059–1070. doi: 10.1002/acp.4104

[ref48] Frigeri T. Paglioli E. de Oliveira E. Rhoton A. L. (2015). Microsurgical anatomy of the central lobe. J. Neurosurg. 122, 483–498. doi: 10.3171/2014.11.JNS14315 25555079

[ref49] Fuchs E. Flügge G. (2014). Adult neuroplasticity: more than 40 years of research. Neural Plast. 2014, 1–10. doi: 10.1155/2014/541870, PMID: 24883212 PMC4026979

[ref50] Fujioka A. Fujioka T. Tsuruta R. Izumi T. Kasaoka S. Maekawa T. (2011). Effects of a constant light environment on hippocampal neurogenesis and memory in mice. Neurosci. Lett. 488, 41–44. doi: 10.1016/j.neulet.2010.11.001, PMID: 21056622

[ref51] Gale S. D. Erickson L. D. Anderson J. E. Brown B. L. Hedges D. W. (2020). Association between exposure to air pollution and prefrontal cortical volume in adults: a cross-sectional study from the UK biobank. Environ. Res. 185:109365. doi: 10.1016/j.envres.2020.109365 32222630

[ref52] Gassar A. A. A. Cha S. H. (2020). Energy prediction techniques for large-scale buildings towards a sustainable built environment: a review. Energ. Buildings 224:110238. doi: 10.1016/j.enbuild.2020.110238

[ref53] Gould van Praag C. D. Garfinkel S. N. Sparasci O. Mees A. Philippides A. O. Ware M.. (2017). Mind-wandering and alterations to default mode network connectivity when listening to naturalistic versus artificial sounds. Sci. Rep. 7:45273. doi: 10.1038/srep45273 28345604 PMC5366899

[ref54] Griesbauer E. M. Manley E. Wiener J. M. Spiers H. J. (2022). London taxi drivers: a review of neurocognitive studies and an exploration of how they build their cognitive map of London. Hippocampus 32, 3–20. doi: 10.1002/hipo.23395, PMID: 34914151

[ref55] Gu Y. Beato J. M. Amarante E. Chesebro A. G. Manly J. J. Schupf N.. (2020). Assessment of leisure time physical activity and brain health in a multiethnic cohort of older adults. JAMA Netw. Open 3, –e2026506. doi: 10.1001/jamanetworkopen.2020.26506, PMID: 33211111 PMC7677758

[ref56] Hahad O. Prochaska J. H. Daiber A. Muenzel T. (2019). Environmental noise-induced effects on stress hormones, oxidative stress, and vascular dysfunction: key factors in the relationship between cerebrocardiovascular and psychological disorders. Oxidative Med. Cell. Longev. 2019, 1–13. doi: 10.1155/2019/4623109 PMC6878772 31814877

[ref57] Harris J. C. Liuzzi M. T. Cardenas-Iniguez C. Larson C. L. Lisdahl K. M. (2023). Gray space and default mode network-amygdala connectivity. Front. Hum. Neurosci. 17:1167786. doi: 10.3389/fnhum.2023.1167786, PMID: 37711221 PMC10498535

[ref58] Hattori S. Hashimoto R. Miyakawa T. Yamanaka H. Maeno H. Wada K.. (2007). Enriched environments influence depression-related behavior in adult mice and the survival of newborn cells in their hippocampi. Behav. Brain Res. 180, 69–76. doi: 10.1016/j.bbr.2007.02.036 17397941

[ref59] He X. Zheng Z. Yang J. Su Y. Wang T. Strnadel B. (2020). Feasibility of incorporating autoclaved aerated concrete waste for cement replacement in sustainable building materials. J. Clean. Prod. 250:119455. doi: 10.1016/j.jclepro.2019.119455

[ref60] Helfrich-Förster C. (2003). The neuroarchitecture of the circadian clock in the brain of *Drosophila melanogaster*. Microsc. Res. Tech. 62, 94–102. doi: 10.1002/jemt.10357 12966496

[ref61] Huntsman D. D. Bulaj G. (2022). Healthy dwelling: design of biophilic interior environments fostering self-care practices for people living with migraines, chronic pain, and depression. Int. J. Environ. Res. Public Health 19:2248. doi: 10.3390/ijerph19042248, PMID: 35206441 PMC8871637

[ref62] Hüttenrauch M. Salinas G. Wirths O. (2016). Effects of long-term environmental enrichment on anxiety, memory, hippocampal plasticity and overall brain gene expression in C57BL6 mice. Front. Mol. Neurosci. 9:62. doi: 10.3389/fnmol.2016.00062 27536216 PMC4971077

[ref63] Ikegame T. Bundo M. Murata Y. Kasai K. Kato T. Iwamoto K. (2013). DNA methylation of the BDNF gene and its relevance to psychiatric disorders. J. Hum. Genet. 58, 434–438. doi: 10.1038/jhg.2013.65 23739121

[ref64] Janak P. H. Tye K. M. (2015). From circuits to behaviour in the amygdala. Nature 517, 284–292. doi: 10.1038/nature14188, PMID: 25592533 PMC4565157

[ref65] Kanai M. Kamiizawa R. Hitora-Imamura N. Minami M. (2022). Exposure to hot and cold environments activates neurons projecting from the paraventricular thalamic nucleus to brain regions related to approach and avoidance behaviors. J. Therm. Biol. 103:103157. doi: 10.1016/j.jtherbio.2021.103157, PMID: 35027193

[ref66] Keller T. A. Just M. A. (2016). Structural and functional neuroplasticity in human learning of spatial routes. NeuroImage 125, 256–266. doi: 10.1016/j.neuroimage.2015.10.015, PMID: 26477660

[ref67] Kempermann G. (2019). Environmental enrichment, new neurons and the neurobiology of individuality. Nat. Rev. Neurosci. 20, 235–245. doi: 10.1038/s41583-019-0120-x, PMID: 30723309

[ref68] Kempermann G. Brandon E. P. Gage F. H. (1998a). Environmental stimulation of 129/SvJ mice causes increased cell proliferation and neurogenesis in the adult dentate gyrus. Curr. Biol. 8, 939–944. doi: 10.1016/S0960-9822(07)00377-6, PMID: 9707406

[ref69] Kempermann G. Kuhn H. G. Gage F. H. (1997). More hippocampal neurons in adult mice living in an enriched environment. Nature 386, 493–495. doi: 10.1038/386493a0, PMID: 9087407

[ref70] Kempermann G. Kuhn H. G. Gage F. H. (1998b). Experience-induced neurogenesis in the senescent dentate gyrus. J. Neurosci. 18, 3206–3212. doi: 10.1523/JNEUROSCI.18-09-03206.1998 9547229 PMC6792643

[ref71] Kempermann G. Song H. Gage F. H. (2015). Neurogenesis in the adult hippocampus. Cold Spring Harb. Perspect. Biol. 7:a018812. doi: 10.1101/cshperspect.a018812, PMID: 26330519 PMC4563705

[ref72] Kentros C. G. Agnihotri N. T. Streater S. Hawkins R. D. Kandel E. R. (2004). Increased attention to spatial context increases both place field stability and spatial memory. Neuron 42, 283–295. doi: 10.1016/S0896-6273(04)00192-8, PMID: 15091343

[ref73] Khalil M. H. (2024). Environmental enrichment: a systematic review on the effect of a changing spatial complexity on hippocampal neurogenesis and plasticity in rodents, with considerations for translation to urban and built environments for humans. Front. Neurosci. 18:1368411. doi: 10.3389/fnins.2024.1368411, PMID: 38919908 PMC11196820

[ref74] Killgore W. D. Vanuk J. R. Dailey N. S. (2022). Treatment with morning blue light increases left amygdala volume and sleep duration among individuals with posttraumatic stress disorder. Front. Behav. Neurosci. 16:910239. doi: 10.3389/fnbeh.2022.910239, PMID: 36172470 PMC9510679

[ref75] Ko J. Sohn J. Noh Y. Koh S. B. Lee S. K. Kim S. Y.. (2024). Effects of ambient air pollution on brain cortical thickness and subcortical volume: a longitudinal neuroimaging study. Neuroepidemiology, 1–11. doi: 10.1159/000539467, PMID: 38815551

[ref76] Kobayashi H. Ikei H. Song C. Kagawa T. Miyazaki Y. (2021). Comparing the impact of forest walking and forest viewing on psychological states. Urban For. Urban Green. 57:126920. doi: 10.1016/j.ufug.2020.126920

[ref77] Kobayashi H. Song C. Ikei H. Park B. J. Kagawa T. Miyazaki Y. (2019). Combined effect of walking and forest environment on salivary cortisol concentration. Front. Public Health 7:480661. doi: 10.3389/fpubh.2019.00376 PMC6920124 31921741

[ref78] Kobayashi H. Song C. Ikei H. Park B. J. Lee J. Kagawa T.. (2017). Population-based study on the effect of a forest environment on salivary cortisol concentration. Int. J. Environ. Res. Public Health 14:931. doi: 10.3390/ijerph14080931, PMID: 28820452 PMC5580633

[ref79] Kojima D. Nakamura T. Banno M. Umemoto Y. Kinoshita T. Ishida Y.. (2018). Head-out immersion in hot water increases serum BDNF in healthy males. Int. J. Hyperth. 34, 834–839. doi: 10.1080/02656736.2017.1394502, PMID: 29157042

[ref80] Kokubun K. Nemoto K. Yamakawa Y. (2024). Continuous inhalation of essential oil increases gray matter volume. Brain Res. Bull. 208:110896. doi: 10.1016/j.brainresbull.2024.110896 38331299

[ref81] Kolarik B. S. Stark S. M. Stark C. E. (2020). Enriching hippocampal memory function in older adults through real-world exploration. Front. Aging Neurosci. 12:158. doi: 10.3389/fnagi.2020.00158, PMID: 32581768 PMC7286388

[ref82] Kolb B. Gibb R. (2015). Plasticity in the prefrontal cortex of adult rats. Front. Cell. Neurosci. 9:15. doi: 10.3389/fncel.2015.00015 25691857 PMC4315042

[ref83] Koyama Y. Mukuda T. Hamasaki S. Nakane H. Kaidoh T. (2018). Short-term heat exposure promotes hippocampal neurogenesis via activation of angiotensin II type 1 receptor in adult rats. Neuroscience 385, 121–132. doi: 10.1016/j.neuroscience.2018.05.045, PMID: 29902505

[ref84] Krech D. Rosenzweig M. R. Bennett E. L. (1960). Effects of environmental complexity and training on brain chemistry. J. Comp. Physiol. Psychol. 53, 509–519. doi: 10.1037/h0045402 13754181

[ref85] Kühn S. Banaschewski T. Bokde A. L. Büchel C. Quinlan E. B. Desrivières S.. (2023a). Trees for brains: current residential tree cover density and its association with brain structure in young adults. J. Environ. Psychol. 89:102047. doi: 10.1016/j.jenvp.2023.102047

[ref86] Kühn S. Düzel S. Eibich P. Krekel C. Wüstemann H. Kolbe J.. (2017). In search of features that constitute an “enriched environment” in humans: associations between geographical properties and brain structure. Sci. Rep. 7:11920. doi: 10.1038/s41598-017-12046-7, PMID: 28931835 PMC5607225

[ref87] Kühn S. Düzel S. Mascherek A. Eibich P. Krekel C. Kolbe J.. (2021). Urban green is more than the absence of city: structural and functional neural basis of urbanicity and green space in the neighbourhood of older adults. Landsc. Urban Plan. 214:104196. doi: 10.1016/j.landurbplan.2021.104196

[ref88] Kühn S. Mascherek A. Filevich E. Lisofsky N. Becker M. Butler O.. (2022). Spend time outdoors for your brain–an in-depth longitudinal MRI study. World J. Biol. Psychiatry 23, 201–207. doi: 10.1080/15622975.2021.1938670, PMID: 34231438

[ref89] Kühn S. Schmalen K. Beijers R. Tyborowska A. Roelofs K. Weerth C. D. (2023b). Green is not the same as green: differentiating between the Association of Trees and Open Green Spaces with Children’s brain structure in the Netherlands. Environ. Behav. 55, 311–334. doi: 10.1177/00139165231183095

[ref90] Kulvicius T. Tamosiunaite M. Ainge J. Dudchenko P. Wörgötter F. (2008). Odor supported place cell model and goal navigation in rodents. J. Comput. Neurosci. 25, 481–500. doi: 10.1007/s10827-008-0090-x, PMID: 18431616 PMC3085777

[ref91] Kuzumaki N. Ikegami D. Tamura R. Hareyama N. Imai S. Narita M.. (2011). Hippocampal epigenetic modification at the brain-derived neurotrophic factor gene induced by an enriched environment. Hippocampus 21, 127–132. doi: 10.1002/hipo.20775, PMID: 20232397

[ref92] Lambert K. Hyer M. Bardi M. Rzucidlo A. Scott S. Terhune-Cotter B.. (2016). Natural-enriched environments lead to enhanced environmental engagement and altered neurobiological resilience. Neuroscience 330, 386–394. doi: 10.1016/j.neuroscience.2016.05.037 27238894

[ref93] Lammer L. Beyer F. Luppa M. Sanders C. Baber R. Engel C.. (2023). Impact of social isolation on grey matter structure and cognitive functions: a population-based longitudinal neuroimaging study. eLife 12:e83660. doi: 10.7554/eLife.83660, PMID: 37337666 PMC10281670

[ref94] Landry T. Huang H. (2021). Mini review: the relationship between energy status and adult hippocampal neurogenesis. Neurosci. Lett. 765:136261. doi: 10.1016/j.neulet.2021.136261, PMID: 34562518 PMC8572164

[ref95] Lang X. Wang Z. Tian X. Wu Y. Zhu S. Liu W. (2022). The effects of extreme high indoor temperature on EEG during a low intensity activity. Build. Environ. 219:109225. doi: 10.1016/j.buildenv.2022.109225

[ref96] Lavenda-Grosberg D. Lalzar M. Leser N. Yaseen A. Malik A. Maroun M.. (2022). Acute social isolation and regrouping cause short-and long-term molecular changes in the rat medial amygdala. Mol. Psychiatry 27, 886–895. doi: 10.1038/s41380-021-01342-4, PMID: 34650208 PMC8515782

[ref97] Leal-Galicia P. Castañeda-Bueno M. Quiroz-Baez R. Arias C. (2008). Long-term exposure to environmental enrichment since youth prevents recognition memory decline and increases synaptic plasticity markers in aging. Neurobiol. Learn. Mem. 90, 511–518. doi: 10.1016/j.nlm.2008.07.005, PMID: 18675926

[ref98] Lederbogen F. Kirsch P. Haddad L. Streit F. Tost H. Schuch P.. (2011). City living and urban upbringing affect neural social stress processing in humans. Nature 474, 498–501. doi: 10.1038/nature10190 21697947

[ref99] LeDoux J. E. (2000). Emotion circuits in the brain. Annu. Rev. Neurosci. 23, 155–184. doi: 10.1146/annurev.neuro.23.1.155 10845062

[ref100] Lee S. Charles S. T. Almeida D. M. (2021). Change is good for the brain: activity diversity and cognitive functioning across adulthood. J. Gerontol. 76, 1036–1048. doi: 10.1093/geronb/gbaa020, PMID: 32025733 PMC8200355

[ref101] Leger M. Paizanis E. Dzahini K. Quiedeville A. Bouet V. Cassel J. C.. (2015). Environmental enrichment duration differentially affects behavior and neuroplasticity in adult mice. Cereb. Cortex 25, 4048–4061. doi: 10.1093/cercor/bhu119, PMID: 24904072

[ref102] Leuner B. Gould E. (2010). Structural plasticity and hippocampal function. Annu. Rev. Psychol. 61, 111–140. doi: 10.1146/annurev.psych.093008.100359, PMID: 19575621 PMC3012424

[ref103] Leung M. K. Lau W. K. Chan C. C. Wong S. S. Fung A. L. Lee T. M. (2018). Meditation-induced neuroplastic changes in amygdala activity during negative affective processing. Soc. Neurosci. 13, 277–288. doi: 10.1080/17470919.2017.1311939, PMID: 28393652

[ref104] Li H. Cui F. Wang T. Wang W. Zhang D. (2024). The impact of sunlight exposure on brain structural markers in the UK biobank. Sci. Rep. 14:10313. doi: 10.1038/s41598-024-59633-z, PMID: 38705875 PMC11070413

[ref105] Liang C. Subramaniam P. Mohd Ridzwan Goh N. S. Kok Wai T. Moustafa A. A. (2023). Digital device use, risk of cognitive impairment, and cognition in healthy older adults: the role of cognitive reserve. Healthcare 11:2822. doi: 10.3390/healthcare11212822 37957967 PMC10649017

[ref106] Lövdén M. Schaefer S. Noack H. Bodammer N. C. Kühn S. Heinze H. J.. (2012). Spatial navigation training protects the hippocampus against age-related changes during early and late adulthood. Neurobiol. Aging 33, 620.e9–620.e22. doi: 10.1016/j.neurobiolaging.2011.02.013 21497950

[ref107] Lövdén M. Schaefer S. Noack H. Kanowski M. Kaufmann J. Tempelmann C.. (2011). Performance-related increases in hippocampal N-acetylaspartate (NAA) induced by spatial navigation training are restricted to BDNF Val homozygotes. Cereb. Cortex 21, 1435–1442. doi: 10.1093/cercor/bhq230, PMID: 21071619

[ref108] Macpherson H. Teo W. P. Schneider L. A. Smith A. E. (2017). A life-long approach to physical activity for brain health. Front. Aging Neurosci. 9:147. doi: 10.3389/fnagi.2017.00147, PMID: 28588474 PMC5440589

[ref109] Maguire E. A. Frackowiak R. S. Frith C. D. (1997). Recalling routes around London: activation of the right hippocampus in taxi drivers. J. Neurosci. 17, 7103–7110. doi: 10.1523/JNEUROSCI.17-18-07103.1997, PMID: 9278544 PMC6573257

[ref110] Maguire E. A. Gadian D. G. Johnsrude I. S. Good C. D. Ashburner J. Frackowiak R. S.. (2000). Navigation-related structural change in the hippocampi of taxi drivers. Proc. Natl. Acad. Sci. 97, 4398–4403. doi: 10.1073/pnas.070039597, PMID: 10716738 PMC18253

[ref111] Maguire E. A. Woollett K. Spiers H. J. (2006). London taxi drivers and bus drivers: a structural MRI and neuropsychological analysis. Hippocampus 16, 1091–1101. doi: 10.1002/hipo.20233, PMID: 17024677

[ref112] Majrashi N. A. Alyami A. S. Shubayr N. A. Alenezi M. M. Waiter G. D. (2022). Amygdala and subregion volumes are associated with photoperiod and seasonal depressive symptoms: a cross-sectional study in the UK biobank cohort. Eur. J. Neurosci. 55, 1388–1404. doi: 10.1111/ejn.15624, PMID: 35165958 PMC9304295

[ref113] Mannan M. Al-Ghamdi S. G. (2021). Indoor air quality in buildings: a comprehensive review on the factors influencing air pollution in residential and commercial structure. Int. J. Environ. Res. Public Health 18:3276. doi: 10.3390/ijerph18063276, PMID: 33810001 PMC8004912

[ref114] McCormick B. P. Brusilovskiy E. Snethen G. Klein L. Townley G. Salzer M. S. (2022). Getting out of the house: the relationship of venturing into the community and neurocognition among adults with serious mental illness. Psychiatr. Rehabil. J. 45, 18–25. doi: 10.1037/prj0000483, PMID: 33793287

[ref115] McCormick C. Dalton M. A. Zeidman P. Maguire E. A. (2021). Characterising the hippocampal response to perception, construction and complexity. Cortex 137, 1–17. doi: 10.1016/j.cortex.2020.12.018, PMID: 33571913 PMC8048772

[ref116] McGlashan E. M. Poudel G. R. Jamadar S. D. Phillips A. J. Cain S. W. (2021). Afraid of the dark: light acutely suppresses activity in the human amygdala. PLoS One 16:e0252350. doi: 10.1371/journal.pone.0252350, PMID: 34133439 PMC8208532

[ref117] Merabet G. H. Essaaidi M. Haddou M. B. Qolomany B. Qadir J. Anan M.. (2021). Intelligent building control systems for thermal comfort and energy-efficiency: a systematic review of artificial intelligence-assisted techniques. Renew. Sust. Energ. Rev. 144:110969. doi: 10.1016/j.rser.2021.110969

[ref118] Migueles J. H. Cadenas-Sanchez C. Esteban-Cornejo I. Torres-Lopez L. V. Aadland E. Chastin S. F.. (2020). Associations of objectively-assessed physical activity and sedentary time with hippocampal gray matter volume in children with overweight/obesity. J. Clin. Med. 9:1080. doi: 10.3390/jcm9041080, PMID: 32290290 PMC7231303

[ref119] Min K. D. Kim J. S. Park Y. H. Shin H. Y. Kim C. Seo S. W.. (2021). New assessment for residential greenness and the association with cortical thickness in cognitively healthy adults. Sci. Total Environ. 778:146129. doi: 10.1016/j.scitotenv.2021.146129, PMID: 33714817

[ref120] Mitra R. Jadhav S. McEwen B. S. Vyas A. Chattarji S. (2005). Stress duration modulates the spatiotemporal patterns of spine formation in the basolateral amygdala. Proc. Natl. Acad. Sci. 102, 9371–9376. doi: 10.1073/pnas.0504011102, PMID: 15967994 PMC1166638

[ref121] Mochizuki-Kawai H. Matsuda I. Mochizuki S. (2020). Viewing a flower image provides automatic recovery effects after psychological stress. J. Environ. Psychol. 70:101445. doi: 10.1016/j.jenvp.2020.101445

[ref122] Moitra M. Owens S. Hailemariam M. Wilson K. S. Mensa-Kwao A. Gonese G.. (2023). Global mental health: where we are and where we are going. Curr. Psychiatry Rep. 25, 301–311. doi: 10.1007/s11920-023-01426-8, PMID: 37256471 PMC10230139

[ref123] Molendijk M. L. Haffmans J. P. Bus B. A. Spinhoven P. Penninx B. W. Prickaerts J.. (2012). Serum BDNF concentrations show strong seasonal variation and correlations with the amount of ambient sunlight. PLoS One 7:e48046. doi: 10.1371/journal.pone.0048046 23133609 PMC3487856

[ref124] Mychasiuk R. Zahir S. Schmold N. Ilnytskyy S. Kovalchuk O. Gibb R. (2012). Parental enrichment and offspring development: modifications to brain, behavior and the epigenome. Behav. Brain Res. 228, 294–298. doi: 10.1016/j.bbr.2011.11.036, PMID: 22173001

[ref125] Nayak M. Das D. Pradhan J. Ahmed R. G. Laureano-Melo R. Dandapat J. (2022). Epigenetic signature in neural plasticity: the journey so far and journey ahead. Heliyon 8:e12292. doi: 10.1016/j.heliyon.2022.e12292 36590572 PMC9798197

[ref126] Nilsson M. Perfilieva E. Johansson U. Orwar O. Eriksson P. S. (1999). Enriched environment increases neurogenesis in the adult rat dentate gyrus and improves spatial memory. J. Neurobiol. 39, 569–578. doi: 10.1002/(SICI)1097-4695(19990615)39:4<569::AID-NEU10>3.0.CO;2-F, PMID: 10380078

[ref127] Nishant R. Kennedy M. Corbett J. (2020). Artificial intelligence for sustainability: challenges, opportunities, and a research agenda. Int. J. Inf. Manag. 53:102104. doi: 10.1016/j.ijinfomgt.2020.102104

[ref128] O'Keefe J. Dostrovsky J. (1971). The hippocampus as a spatial map: preliminary evidence from unit activity in the freely-moving rat. Brain Res. 34, 171–175. doi: 10.1016/0006-8993(71)90358-1, PMID: 5124915

[ref129] Olajiga O. K. Ani E. C. Sikhakane Z. Q. Olatunde T. M. (2024). A comprehensive review of energy-efficient lighting technologies and trends. Eng. Sci. Technol. J. 5, 1097–1111. doi: 10.51594/estj.v5i3.973

[ref082] Özer F. S. (2021). Neuroscience for understanding and developing sustainability: neurosustainability. J. Bus. Innovat. Govern. 4, 132–148. doi: 10.54472/jobig.948854

[ref130] Park S. A. Lee A. Y. Park H. G. Lee W. L. (2019). Benefits of gardening activities for cognitive function according to measurement of brain nerve growth factor levels. Int. J. Environ. Res. Public Health 16:760. doi: 10.3390/ijerph16050760 30832372 PMC6427672

[ref131] Pascual-Leone A. Amedi A. Fregni F. Merabet L. B. (2005). The plastic human brain cortex. Annu. Rev. Neurosci. 28, 377–401. doi: 10.1146/annurev.neuro.27.070203.144216 16022601

[ref132] Pavlov K. I. Mukhin V. N. Kamenskaya V. G. Klimenko V. M. (2016). Dependence of cerebral-cortex activation in women on environmental factors. Izvestiya Atmospheric Oceanic Phys. 52, 737–744. doi: 10.1134/S0001433816070045

[ref133] Pearce M. T. Zaidel D. W. Vartanian O. Skov M. Leder H. Chatterjee A.. (2016). Neuroaesthetics: the cognitive neuroscience of aesthetic experience. Perspect. Psychol. Sci. 11, 265–279. doi: 10.1177/1745691615621274 26993278

[ref134] Pham A. D. Ngo N. T. Truong T. T. H. Huynh N. T. Truong N. S. (2020). Predicting energy consumption in multiple buildings using machine learning for improving energy efficiency and sustainability. J. Clean. Prod. 260:121082. doi: 10.1016/j.jclepro.2020.121082

[ref135] Phelps E. A. LeDoux J. E. (2005). Contributions of the amygdala to emotion processing: from animal models to human behavior. Neuron 48, 175–187. doi: 10.1016/j.neuron.2005.09.025 16242399

[ref136] Pineda J. C. D. Kokubun K. Ikaga T. Yamakawa Y. (2021). Housing quality and behavior affect brain health and anxiety in healthy Japanese adults. Sci. Rep. 11:11999. doi: 10.1038/s41598-021-91363-4, PMID: 34099762 PMC8184752

[ref137] Poucet B. Hok V. (2017). Remembering goal locations. Curr. Opin. Behav. Sci. 17, 51–56. doi: 10.1016/j.cobeha.2017.06.003

[ref138] Priestley J. B. Bowler J. C. Rolotti S. V. Fusi S. Losonczy A. (2022). Signatures of rapid plasticity in hippocampal CA1 representations during novel experiences. Neuron 110, 1978–1992.e6. doi: 10.1016/j.neuron.2022.03.026, PMID: 35447088 PMC9233041

[ref139] Rojas-Carvajal M. Sequeira-Cordero A. Brenes J. C. (2022). The environmental enrichment model revisited: a translatable paradigm to study the stress of our modern lifestyle. Eur. J. Neurosci. 55, 2359–2392. doi: 10.1111/ejn.15160, PMID: 33638921

[ref140] Roozendaal B. McEwen B. S. Chattarji S. (2009). Stress, memory and the amygdala. Nat. Rev. Neurosci. 10, 423–433. doi: 10.1038/nrn2651 19469026

[ref141] Sahay A. Scobie K. N. Hill A. S. O'Carroll C. M. Kheirbek M. A. Burghardt N. S.. (2011). Increasing adult hippocampal neurogenesis is sufficient to improve pattern separation. Nature 472, 466–470. doi: 10.1038/nature09817, PMID: 21460835 PMC3084370

[ref142] Santoso D. I. I. Yolanda S. Redjeki S. Andraini T. Ivanali K. (2020). Continuous environmental enrichment and aerobic exercise improves spatial memory: focus on rat hippocampal BDNF and NGF. Compar. Exerc. Physiol. 16, 121–128.

[ref143] Scaplen K. M. Gulati A. A. Heimer-McGinn V. L. Burwell R. D. (2014). Objects and landmarks: hippocampal place cells respond differently to manipulations of visual cues depending on size, perspective, and experience. Hippocampus 24, 1287–1299. doi: 10.1002/hipo.22331, PMID: 25045010 PMC5615844

[ref144] Schröder J. K. Abdel-Hafiz L. Ali A. A. Cousin T. C. Hallenberger J. Rodrigues Almeida F.. (2023). Effects of the light/dark phase and constant light on spatial working memory and spine plasticity in the mouse Hippocampus. Cells 12:1758. doi: 10.3390/cells12131758, PMID: 37443792 PMC10340644

[ref145] Segovia G. Yagüe A. G. García-Verdugo J. M. Mora F. (2006). Environmental enrichment promotes neurogenesis and changes the extracellular concentrations of glutamate and GABA in the hippocampus of aged rats. Brain Res. Bull. 70, 8–14. doi: 10.1016/j.brainresbull.2005.11.005, PMID: 16750477

[ref146] Shin J. Lee H. W. Jin S. W. Lee I. (2022). Subtle visual change in a virtual environment induces heterogeneous remapping systematically in CA1, but not CA3. Cell Rep. 41:111823. doi: 10.1016/j.celrep.2022.111823 36516763

[ref147] Shin N. Rodrigue K. M. Yuan M. Kennedy K. M. (2024). Geospatial environmental complexity, spatial brain volume, and spatial behavior across the Alzheimer's disease spectrum. Alzheimer's Dement. 16:e12551. doi: 10.1002/dad2.12551 PMC10883241 38390561

[ref148] Simon K. R. Merz E. C. He X. Noble K. G. (2022). Environmental noise, brain structure, and language development in children. Brain Lang. 229:105112. doi: 10.1016/j.bandl.2022.105112, PMID: 35398600 PMC9126644

[ref149] Singh S. Sharma P. K. Yoon B. Shojafar M. Cho G. H. Ra I. H. (2020). Convergence of blockchain and artificial intelligence in IoT network for the sustainable smart city. Sustain. Cities Soc. 63:102364. doi: 10.1016/j.scs.2020.102364

[ref150] Skillington K. Crawford R. H. Warren-Myers G. Davidson K. (2022). A review of existing policy for reducing embodied energy and greenhouse gas emissions of buildings. Energy Policy 168:112920. doi: 10.1016/j.enpol.2022.112920

[ref151] Smith O. V. Penhale S. H. Ott L. R. Rice D. L. Coutant A. T. Glesinger R.. (2024). Everyday home radon exposure is associated with altered structural brain morphology in youths. Neurotoxicology 102, 114–120. doi: 10.1016/j.neuro.2024.04.007, PMID: 38703899 PMC11139553

[ref152] Son J. Y. Choi H. M. Fong K. C. Heo S. Lim C. C. Bell M. L. (2021). The roles of residential greenness in the association between air pollution and health: a systematic review. Environ. Res. Lett. 16:093001. doi: 10.1088/1748-9326/ac0e61 PMC12363650 40837388

[ref153] Soni A. Das P. K. Hashmi A. W. Yusuf M. Kamyab H. Chelliapan S. (2022). Challenges and opportunities of utilizing municipal solid waste as alternative building materials for sustainable development goals: a review. Sustain. Chem. Pharm. 27:100706. doi: 10.1016/j.scp.2022.100706

[ref154] Squire L. R. Genzel L. Wixted J. T. Morris R. G. (2015). Memory consolidation. Cold Spring Harb. Perspect. Biol. 7:a021766. doi: 10.1101/cshperspect.a021766, PMID: 26238360 PMC4526749

[ref155] Stobbe E. Lorenz R. C. Kühn S. (2023). On how natural and urban soundscapes alter brain activity during cognitive performance. J. Environ. Psychol. 91:102141. doi: 10.1016/j.jenvp.2023.102141

[ref156] Sudimac S. Kühn S. (2022). A one-hour walk in nature reduces amygdala activity in women, but not in men. Front. Psychol. 13:931905. doi: 10.3389/fpsyg.2022.931905, PMID: 36248579 PMC9556704

[ref157] Sudimac S. Kühn S. (2024). Can a nature walk change your brain? Investigating hippocampal brain plasticity after one hour in a forest. Investigating Hippocampal Brain Plasticity After One Hour in a Forest. 10.1016/j.envres.2024.119813 39155041

[ref158] Sudimac S. Sale V. Kühn S. (2022). How nature nurtures: amygdala activity decreases as the result of a one-hour walk in nature. Mol. Psychiatry 27, 4446–4452. doi: 10.1038/s41380-022-01720-6, PMID: 36059042 PMC9734043

[ref159] Sun Y. Ma D. Jiang Z. Han Q. Liu Y. Chen G. (2024). The causal relationship between physical activity, sedentary behavior and brain cortical structure: a Mendelian randomization study. Cereb. Cortex 34:bhae119. doi: 10.1093/cercor/bhae119 38566508

[ref160] Sweatt J. D. (2013). The emerging field of neuroepigenetics. Neuron 80, 624–632. doi: 10.1016/j.neuron.2013.10.023, PMID: 24183015 PMC3878295

[ref161] Tani Y. Fujiwara T. Sugihara G. Hanazato M. Suzuki N. Machida M.. (2022). Neighborhood beauty and the brain in older Japanese adults. Int. J. Environ. Res. Public Health 20:679. doi: 10.3390/ijerph20010679, PMID: 36612997 PMC9819975

[ref162] Tardif C. L. Gauthier C. J. Steele C. J. Bazin P. L. Schäfer A. Schaefer A.. (2016). Advanced MRI techniques to improve our understanding of experience-induced neuroplasticity. NeuroImage 131, 55–72. doi: 10.1016/j.neuroimage.2015.08.047 26318050

[ref163] Urban-Wojcik E. J. Lee S. Grupe D. W. Quinlan L. Gresham L. Hammond A.. (2022). Diversity of daily activities is associated with greater hippocampal volume. Cogn. Affect. Behav. Neurosci. 22, 1–13. doi: 10.3758/s13415-021-00942-5 34599488 PMC8792192

[ref164] Uy J. P. Goldenberg D. Tashjian S. M. Do K. T. Galván A. (2019). Physical home environment is associated with prefrontal cortical thickness in adolescents. Dev. Sci. 22:e12834. doi: 10.1111/desc.12834, PMID: 30964952

[ref165] Varma V. R. Chuang Y. F. Harris G. C. Tan E. J. Carlson M. C. (2015). Low-intensity daily walking activity is associated with hippocampal volume in older adults. Hippocampus 25, 605–615. doi: 10.1002/hipo.22397, PMID: 25483019 PMC4425252

[ref166] Villemonteix T. Guerreri M. Deantoni M. Balteau E. Schmidt C. Stee W.. (2023). Sleep-dependent structural neuroplasticity after a spatial navigation task: a diffusion imaging study. J. Neurosci. Res. 101, 1031–1043. doi: 10.1002/jnr.25176, PMID: 36787426

[ref167] Vyas A. Mitra R. Rao B. S. Chattarji S. (2002). Chronic stress induces contrasting patterns of dendritic remodeling in hippocampal and amygdaloid neurons. J. Neurosci. 22, 6810–6818. doi: 10.1523/JNEUROSCI.22-15-06810.2002, PMID: 12151561 PMC6758130

[ref168] Walker W. H. Borniger J. C. Gaudier-Diaz M. M. Hecmarie Meléndez-Fernández O. Pascoe J. L. Courtney DeVries A.. (2020). Acute exposure to low-level light at night is sufficient to induce neurological changes and depressive-like behavior. Mol. Psychiatry 25, 1080–1093. doi: 10.1038/s41380-019-0430-4, PMID: 31138889 PMC6881534

[ref169] Wang R. Xu X. Li Y. Zhou C. Zhao J. (2019). Effect of perceived novelty on aesthetic preference. Proc. Instit. Civil Eng. Urban Design Plann. 172, 102–110. doi: 10.1680/jurdp.18.00046

[ref170] Wenger E. Schaefer S. Noack H. Kühn S. Mårtensson J. Heinze H. J.. (2012). Cortical thickness changes following spatial navigation training in adulthood and aging. NeuroImage 59, 3389–3397. doi: 10.1016/j.neuroimage.2011.11.015, PMID: 22108645

[ref171] Wijesooriya N. Brambilla A. Markauskaite L. (2023). Biophilic design frameworks: a review of structure, development techniques and their compatibility with LEED sustainable design criteria. Clean. Product. Lett. 4:100033. doi: 10.1016/j.clpl.2023.100033

[ref172] Wolff T. Iyer N. A. Rubin G. M. (2015). Neuroarchitecture and neuroanatomy of the Drosophila central complex: a GAL4-based dissection of protocerebral bridge neurons and circuits. J. Comp. Neurol. 523, 997–1037. doi: 10.1002/cne.23705, PMID: 25380328 PMC4407839

[ref173] Wolters N. E. Mobach L. Wuthrich V. M. Vonk P. Van der Heijde C. M. Wiers R. W.. (2023). Emotional and social loneliness and their unique links with social isolation, depression and anxiety. J. Affect. Disord. 329, 207–217. doi: 10.1016/j.jad.2023.02.096, PMID: 36842647

[ref174] Wu M. H. Ng T. S. Skitmore M. R. (2016). Sustainable building envelope design by considering energy cost and occupant satisfaction. Energy Sustain. Dev. 31, 118–129. doi: 10.1016/j.esd.2015.12.003

[ref175] Yao B. Christian K. M. He C. Jin P. Ming G. L. Song H. (2016). Epigenetic mechanisms in neurogenesis. Nat. Rev. Neurosci. 17, 537–549. doi: 10.1038/nrn.2016.70, PMID: 27334043 PMC5610421

[ref176] Yuan A. Halabicky O. Rao H. Liu J. (2023). Lifetime air pollution exposure, cognitive deficits, and brain imaging outcomes: a systematic review. Neurotoxicology 96, 69–80. doi: 10.1016/j.neuro.2023.03.006, PMID: 37001821 PMC10963081

[ref177] Yuliana Y. (2021). Amygdala changes through breathing exercise in coping with the COVID-19 pandemic. Int. J. Res. STEM Educ. 3, 07–16. doi: 10.31098/ijrse.v3i1.457

[ref178] Zhang S. Manahan-Vaughan D. (2015). Spatial olfactory learning contributes to place field formation in the hippocampus. Cereb. Cortex 25, 423–432. doi: 10.1093/cercor/bht239 24008582 PMC4380081

[ref179] Zhang Y. Zhu M. Sun Y. Tang B. Zhang G. An P.. (2021). Environmental noise degrades hippocampus-related learning and memory. Proc. Natl. Acad. Sci. 118:e2017841117. doi: 10.1073/pnas.2017841117, PMID: 33229555 PMC7797896

[ref180] Zhong W. Schröder T. Bekkering J. (2022). Biophilic design in architecture and its contributions to health, well-being, and sustainability: a critical review. Front. Architect. Res. 11, 114–141. doi: 10.1016/j.foar.2021.07.006

