# Corrigendum: Neurosustainability

**DOI:** 10.3389/fnhum.2024.1524620

**Published:** 2024-11-22

**Authors:** Mohamed Hesham Khalil

**Affiliations:** Department of Architecture, Faculty of Architecture and History of Art, School of Arts and Humanities, University of Cambridge, Cambridge, United Kingdom

**Keywords:** neuroplasticity, environmental enrichment, nature, cortical thickness, adult hippocampal neurogenesis, amygdala, mental health and modern lifestyles, human brain health

In the published article, the reference Özer, F. Ş. (2021). Neuroscience for understanding and developing sustainability: neurosustainability. *J. Bus. Innovat. Govern*. 4, 132–148. doi: 10.54472/jobig.948854, was not cited in the article. The citation has now been inserted in the references section as well as **Section 3 Neurosustainability: Changes, challenges, chances**, *3.1.2 Neurosustainability and sustainability: plasticity before and through the planet*, as a footnote at the end of paragraph 2, which should read:

“This paper has introduced a novel theory titled “Neurosustainability,” which explores the role of the physical environment—both natural and built—in sustaining neuroplasticity through environmental enrichment strategies. Even though we raise awareness among researchers in sustainability, the word sustainability used in this article refers to the quintessential meaning of the word “sustainability” beyond its applied definition. It has come to the author's attention that the term Neurosustainability was previously utilized in a different context by Özer (2021). This prior usage was not identified during this article's ideation phase or literature review process, as the primary database searches conducted do not include the source containing Özer's work. Özer's paper refers to a different meaning of the term Neurosustainability, however, which does not convey the same meaning introduced in this article and does not infringe its originality. However, as this article raised awareness for researchers in sustainability to be Neurosustainability-conscious, we acknowledge Özer's work as relevant to the awareness raised. This situation underscores the multidisciplinary interest in and relevance of Neurosustainability and sets a foundation for future scholarly discourse to refine and differentiate terminology usage within varied research contexts. Future research may cite only one of the two articles depending on the discipline; therefore, future research may no longer need to cite both articles unless needed.”

The authors apologize for this error and state that this does not change the scientific conclusions of the article in any way. The original article has been updated.

## References

[B1] Özer F. Ş. (2021). Neuroscience for understanding and developing sustainability: neurosustainability. J. Bus. Innovat. Govern. 4, 132–148. 10.54472/jobig.948854

